# Porous Organic Frameworks for Lithium-Metal Anodes: Design Strategies, Mechanisms, and Future Perspectives

**DOI:** 10.3390/nano16120756

**Published:** 2026-06-16

**Authors:** Bozhong Tian, Yibo Wu, Muhammad Ahsan Waseem, Junaid Aslam, Weiwei Sun, Chao Yang

**Affiliations:** Department of Chemical Engineering, School of Environmental and Chemical Engineering, Shanghai University, 99 Shangda Road, Shanghai 200444, China

**Keywords:** covalent organic frameworks, design principles, lithium metal batteries, metal–organic frameworks, porous organic frameworks

## Abstract

Lithium-metal batteries (LMBs) are considered among the most promising high-performance energy storage systems because lithium metal possesses extremely high theoretical capacity and the lowest electrochemical potential among anode materials. However, their practical implementation remains severely limited by several critical challenges at the nanoscale, including uncontrolled lithium dendrite growth, unstable solid-electrolyte interphase formation, low Coulombic Efficiency, and large volume fluctuations during repeated lithium plating and stripping processes. In recent years, nanostructured porous framework materials have emerged as effective host structures and interfacial regulators for stabilizing lithium metal anodes due to their high surface areas, tunable pore architectures, and functionalizable chemical environments. In this review, we systematically summarize the recent progress in metal–organic frameworks (MOFs), covalent organic frameworks (COFs), covalent organic polymers (COPs) and other organic framework materials for lithium-metal anode applications. First, the fundamental working principles of LMBs and the major challenges associated with lithium metal anodes are discussed. Subsequently, the structural characteristics and advantages of MOFs, COFs, COPs and other framework materials are compared, followed by a detailed discussion of lithium storage mechanisms in porous frameworks, including lithium adsorption and nucleation, regulation of plating and stripping, dendrite suppression, and stabilization of the solid electrolyte interphase. Key design strategies, including hierarchical pore engineering, lithiophilic chemical functionalization, and electronic conductivity enhancement, are systematically highlighted. Representative advances in COF-based, MOF-based, and COP-based materials for lithium metal stabilization are critically summarized and compared. Finally, the remaining challenges and future research directions for porous framework materials in LMBs are discussed. This review aims to provide fundamental insights and design strategies for the rational development of advanced porous framework materials toward safe, stable, and high-energy LMBs.

## 1. Introduction

**Figure 1:**
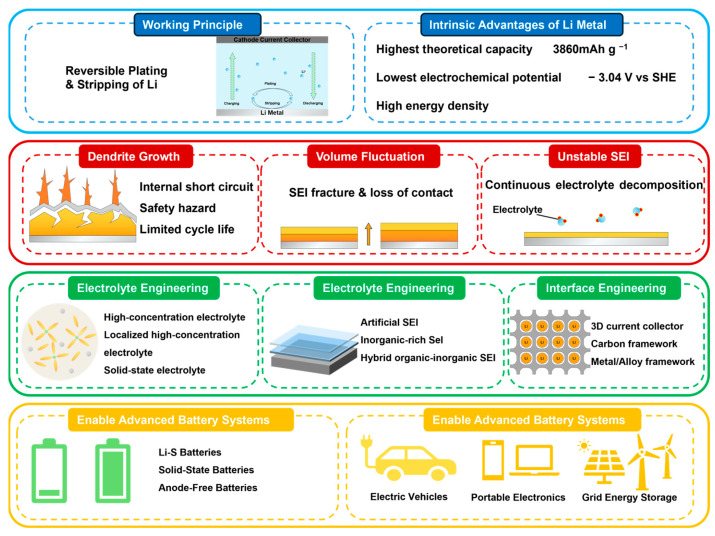
Fundamental of lithium-metal batteries.

Lithium-metal batteries (LMBs) have attracted significant interest as next-generation energy storage systems due to their exceptionally high theoretical energy density. In contrast to conventional lithium ion batteries (LIBs), whose anode materials (graphite or silicon) rely on intercalation mechanisms, in LMBs, metallic lithium is used directly as the anode. Compared with conventional graphite anodes, which are severely constrained by low theoretical specific capacity (372 mAh g^−1^) and fail to meet the urgent demand for next-generation high-energy-density energy storage, lithium metal has the highest theoretical specific capacity and the lowest electrochemical potential; it offers a significant advantage for batteries with higher energy density and better performance. These properties make LMBs promising candidates for advanced applications such as electric vehicles, portable electronics, and large-scale renewable energy storage systems. The basic working principle of LMBs is the reversible plating and stripping of lithium metal during battery operation. During charging, lithium ions (Li^+^) that pass through the electrolyte from the cathode to the anode are reduced at the anode surface, forming metallic lithium. Conversely, during discharge, the deposited lithium metal is oxidized to Li^+^, which migrates back to the cathode via the electrolyte to participate in the electrochemical reaction. This plating and stripping process is very different from the intercalation mechanism in LIBs, in which Li^+^ is inserted into the layered structure of the anode material, as displayed in Figure 1. As lithium metal is not dependent on host materials for ion storage, it allows for much higher energy densities.

Despite their significant benefits, lithium metal anodes (LMAs) have several critical challenges that limit their practical applications. One of the most important problems is lithium dendrite formation. Deposition process of lithium is very sensitive to surface morphology, electrolyte, and current density [1,2,3]. Any non-uniformity in the lithium-ion flux or surface condition can cause unequal deposition of lithium and eventually cause the formation of lithium dendrites. Due to the instability of the local current density and the uneven surface, lithium deposition becomes uneven during repeated charge–discharge cycles. This non-uniform deposition causes dendritic growth of the lithium structure, which worsens during cycling. These dendrites may invade the separator and cause internal short-circuits with serious safety consequences such as overheating or thermal runaway [4,5,6]. Another important aspect of LMB operation is the formation of the solid electrolyte interphase (SEI). When lithium metal is first exposed to the electrolyte, there are spontaneous reactions between the highly reactive lithium surface and electrolyte components. These reactions form a passivation layer, called the SEI, which is key to the battery’s performance. Ideally, the SEI will be ionically conductive, electronically insulating, and mechanically stable, protecting the lithium surface while allowing Li^+^ to pass. However, in practical systems, the SEI layer is fragile and unstable, leading to continuous electrolyte decomposition and capacity loss during repeated cycling [7,8,9,10]. Due to the constantly changing volume of lithium metal during plating and stripping, the SEI layer often cracks and reforms multiple times. This continuous regeneration of SEI consumes both lithium and the electrolyte, and causes poor Coulombic Efficiency (CE) and rapid capacity degradation. The formation of electrically isolated “dead lithium” makes this situation even worse and reduces the amount of active lithium available for electrochemical reaction. In addition, the LMAs exhibit large volume expansion during cycling. The repetitive depositions and dissolutions of the lithium lead to large morphological changes at the electrode surface, which may degrade the electrode structure and battery stability. These structural changes may also impede the SEI layer and promote further dendrite growth, leading to a cycle of degradation that eventually limits battery lifetime [11,12,13].

**Figure 2:**
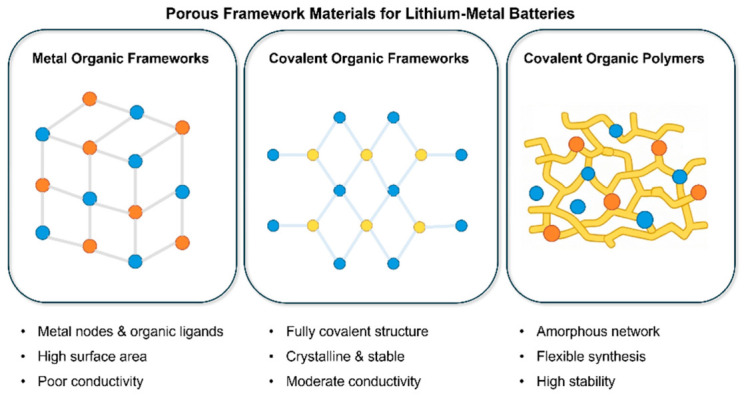
Porous framework materials for lithium-metal batteries.

Therefore, developing effective strategies to control lithium deposition and enhance LMA’s stability is a key challenge in the development of next-generation high-energy batteries. To overcome these difficulties, several approaches have been examined, such as electrolyte engineering, artificial SEI layers, protective coatings, and the development of host materials for lithium deposition. Among these approaches, the design of structured host materials has proven to be an effective method for stabilizing LMAs [14,15,16]. Host materials can provide a conductive framework that accommodates the deposition of lithium, reduces the current density, and suppresses dendrite formation. In particular, porous materials with large surface areas and tunable structures have shown great potential as hosts or scaffolds for lithium metal. Compared to conventional inorganic host materials (such as porous metals, alloys, or rigid inorganic carbon matrices), which typically suffer from a heavy dead weight, structural brittleness, and a lack of molecular-level tailorability, porous organic frameworks (POFs) exhibit distinct advantages. POFs feature a lightweight organic backbone that minimizes the dead mass of the electrode, highly flexible or resilient structures capable of dynamically accommodating volumetric variations, and precisely designable chemical environments that enable the uniform distribution of atomic-scale lithiophilic sites. In recent years, porous framework materials have attracted significant attention for energy storage applications because of their well-defined architecture, large surface areas, and tunable chemical functionalities. Among them, metal–organic frameworks (MOFs), covalent–organic frameworks (COFs) and covalent–organic polymers (COPs) have garnered significant attention. These materials contain well-defined building blocks linked by coordination or covalent bonds and they have a highly porous structure and versatile design strategies, enabling precise control over pore size, surface chemistry, and structural functionality (Figure 2) [17,18]. MOFs are crystalline porous materials that are built from metal ions or metal clusters bonded with organic ligands. MOFs exhibit exceptionally high surface areas and highly ordered pore structures, efficient ion transport, and a high density of active sites for nucleating lithium. Moreover, MOFs can be converted into conductive carbon frameworks via controlled pyrolysis, yielding MOF-derived materials with improved electrical conductivity and structural stability [19,20,21]. These properties make MOF-based materials very attractive candidates to be used as lithium metal hosts for electrode materials in lithium batteries. COFs are another significant class of porous materials that are made of pure light elements like carbon, hydrogen, boron, nitrogen, and oxygen connected by strong covalent bonds. Unlike MOFs, COFs have outstanding chemical and thermal stability as they have fully covalent structures. Ordered and periodic pore channels of COFs can enable rapid ion transport and uniform lithium distribution. In addition, incorporating redox-active functional groups into COF backbones offers opportunities to improve lithium storage performance [22,23,24]. COPs, unlike COFs, are usually amorphous and offer several advantages for battery applications. COPs can be synthesized using versatile polymerization techniques to afford excellent control of chemical composition, functional groups, and porosity. Their structural robustness, chemical stability, and tunable functionality make them attractive materials for regulating lithium nucleation and LMAs’ stability. Furthermore, COP-based materials can be readily combined with conductive carbon materials or metal nanoparticles to enhance electrical conductivity and electrochemical performance [25,26,27]. Such characteristics make MOFs, COFs, COPs and other organic framework materials particularly suitable for electrochemical energy storage systems, including LMBs, for which efficient ion transport, stable electrode structures, and controlled lithium deposition are key to improving battery performance. Their porous structures can accommodate lithium deposition, reduce local current density, and enable uniform lithium plating and stripping. Additionally, introducing heteroatoms and polar functional groups into such frameworks may enhance lithiophilicity, promoting uniform lithium nucleation and reducing dendrite formation [28,29,30,31,32].

Although MOFs, COFs, and COPs share several similarities in their porous nature and structural tunability, they differ significantly in bonding interactions, crystallinity, and chemical stability. Understanding these structural and chemical differences is crucial for designing efficient framework materials for LMAs [33,34]. In this review, we give a comprehensive overview of the recent development of porous framework materials of different types, such as MOFs, COFs, COPs and other framework materials, as advanced anode or host materials for LMBs. The basic challenges of LMAs and the design principles of porous framework materials have also been summarized and discussed. Based on the presentation and comparison of MOF, COF, COP and other framework based materials for LMBs, mechanisms of lithium storage and dendrite suppression in these frameworks have been illuminated. In addition, the design strategies of organic framework materials with enhanced LMBs performances have also been suggested and the remaining barriers and future research directions on the development of porous framework materials in LMBs are highlighted. This review will provide insights into the rational design and future exploration of next-generation porous materials for safe, stable, and high-energy LMBs.

While several recent reviews have discussed the application of porous materials in energy storage, they have predominantly focused on single material classes (e.g., exclusively MOFs or COFs) or specific battery components like separators and cathodes. The novelty of this review lies in its systematic, comparative evaluation of both highly crystalline frameworks (MOFs, COFs) and flexible/amorphous networks (COPs, HOFs) specifically for the interfacial regulation of lithium metal anodes. By holistically contrasting their distinct ion-transport mechanisms, mechanical robustness, and structural degradation behaviors, this work provides unified design strategies—ranging from hierarchical pore engineering to electronic conductivity enhancement—that transcend individual material boundaries to accelerate the development of next-generation safe and high-energy LMBs [35,36,37].

## 2. Lithium Storage Mechanism in Porous Frameworks

**Figure 3:**
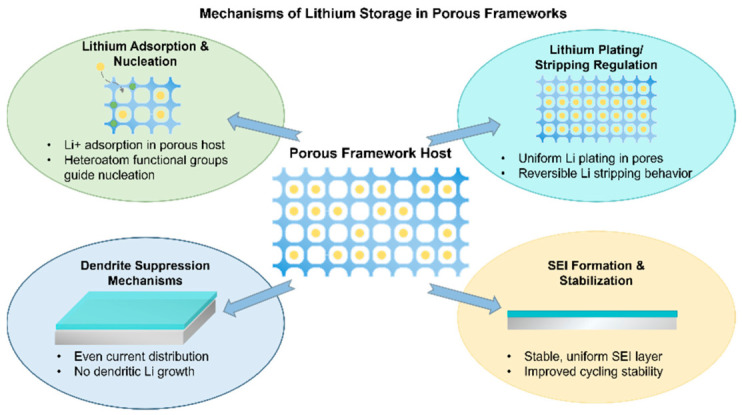
Mechanism of Lithium storage in porous frameworks.

Porous framework materials such as MOFs, COFs, and COPs have emerged as promising hosts for lithium metal due to their unique structural features, including high surface areas, tunable pore structures, and functionalized frameworks. These characteristics allow them to control lithium nucleation, deposition, and transport in the electrode structure. Understanding the mechanisms of lithium storage and deposition in these porous materials is vital for designing stable, high-performance LMBs (Figure 3).

### 2.1. Enhanced Lithium Adsorption and Nucleation

The first step in lithium storage in porous frameworks is the adsorption of Li^+^ on the framework’s internal surfaces. Due to the high specific surface areas and a large number of active sites in porous framework materials, Li^+^ ions can be distributed throughout the porous network. Specifically, density functional theory (DFT) and experimental studies reveal that lithium ions preferentially reside at strongly electronegative heteroatoms (such as pyridinic N, pyrrolic N, or O atoms), electron-rich functional groups (e.g., C=O, C=N), or coordinate directly with open metal sites in MOFs. The dominant storage mechanism in these frameworks differs fundamentally from conventional intercalation into a crystalline lattice; it operates primarily through a surface-driven adsorption and underpotential deposition process. These abundant polar active sites establish strong dipole-cation or cation-π interactions, effectively anchoring the solvated Li^+^ ions, accelerating their desolvation, and subsequently guiding the initial uniform nucleation. The presence of heteroatoms in the framework, such as nitrogen, oxygen, sulfur, or boron, often increases Li^+^ affinity for the host material [38,39,40]. These heteroatoms, which contain functional groups, serve as lithiophilic sites, reducing the nucleation energy barrier for lithium deposition. Uniform nucleation of lithium is important to prevent local lithium accumulation, which can lead to dendrite formation. In porous frameworks, the nucleation sites provided by abundant functional groups and metal centers facilitate homogeneous nucleation of lithium throughout the host structure. As a result, lithium deposition occurs more uniformly within the porous network rather than forming isolated dendritic structures on the electrode surface. This uniform nucleation behavior ensures a much more stable cycling and safer LMBs [41,42,43].

### 2.2. Enhanced Lithium Plating and Stripping Regulation

During charging, Li^+^ is reduced and deposited as metallic lithium within the pores or at the surface of the porous framework. The highly porous architecture of MOFs, COFs, and COPs are of great importance for controlling lithium plating [44]. The interconnected pore channels enable Li^+^ to diffuse efficiently through the host structure, resulting in a uniform distribution of lithium deposition. The large internal surface area of these types of materials also reduces the local current density during lithium plating. Lower current density helps suppress the formation of lithium dendrites, as Li^+^ ions are more evenly deposited across the electrode surface. In addition, the porous framework provides sufficient room to accommodate lithium metal, effectively buffering the volume changes associated with repeated plating and stripping cycles. During discharge, the deposited lithium is oxidized back to Li^+^ and released into the electrolyte. The porous host structure is conducive to this stripping process as it preserves the electrical connection between the deposited lithium and the conduction network. This ensures that lithium can be used reversibly during cycling, reducing the formation of electrically isolated “dead lithium” and improving CE [45,46].

### 2.3. Dendrite Suppression Mechanisms

One of the most important benefits of porous framework hosts is their ability to inhibit lithium dendrite growth. Dendrite formation is usually a result of uneven deposition of lithium and local current density variation. In porous frameworks, multiple mechanisms contribute to the suppression of dendrite growth. First, the high surface area of the host material spreads Li^+^ over a wider area, reducing the likelihood of Li^+^ localization. Second, the interconnected pore structures direct lithium deposition within the internal framework rather than allowing it to accumulate on the external electrode surface. This restricted deposition behavior is an effective solution for preventing the growth of needle-like lithium dendrites [47,48]. Furthermore, the presence of lithiophilic functional groups within the framework promotes uniform nucleation of lithium and stabilizes lithium clusters during deposition. Some MOFs and heteroatom-doped frameworks can also improve ionic conductivity and control the flux of Li^+^, thereby promoting uniform lithium growth. Together, these effects provide a more stable, controlled environment for lithium deposition, thereby significantly improving the safety and cycle life of LMBs.

### 2.4. Solid Electrolyte Interphase Formation and Stabilization

The formation and stability of the solid electrolyte interphase are also highly affected by porous framework materials. In conventional LMAs, the constant formation and dissolution of the SEI layer during cycling cause electrolyte consumption and capacity loss. However, when lithium is deposited within a porous host, the framework can help stabilize the SEI layer. The porous architecture enables deposition of lithium in a confined environment, thus minimizing direct exposure of lithium metal to the electrolyte [49,50]. This confined environment favors the development of a more uniform and stable SEI layer on the internal surfaces of the host material. In addition, the functional groups that are present in MOFs, COFs, and COPs can affect the composition and mechanical stability of the SEI layer. A stable SEI layer enhances Li^+^ transport while preventing continuous electrolyte decomposition. As a result, the CE of LMBs is improved, and the long-term cycling stability is greatly improved [51]. Therefore, the combination of uniform lithium nucleation, controlled plating and stripping behavior, dendrite suppression, and SEI stabilization makes porous framework materials highly effective hosts for lithium metal in advanced battery systems [52]. Porous framework materials such as MOFs, COFs, and COPs provide an effective platform for regulating lithium storage behavior in LMBs. Their high surface area, tunable pore structures, and rich functional groups enable uniform Li adsorption, controlled nucleation, and stable Li plating/stripping. These structural advantages facilitate a reduction in local current density, enable volume changes, and guide lithium deposition within the porous host, thereby suppressing dendrite formation. In addition, the restricted deposition environment within these frameworks helps form a more stable and uniform SEI, thereby improving CE and cycling stability. Therefore, understanding and optimizing lithium storage mechanisms in porous frameworks is highly important for the rational design of advanced host materials that will lead to safer, high-performance LMBs [53,54].

## 3. Application of POF for LMA

Serving primarily as artificial solid electrolyte interphases, 3D structural hosts, POFs provide a highly versatile platform to mitigate the persistent challenges of uncontrolled dendrite growth, uneven ion flux, and severe volume fluctuations. It is worth noting that while certain redox-active POFs can directly function as independent insertion-type anodes or organic cathodes by utilizing their active molecular units for chemical lithium storage, their deployment in the specific context of LMBs is currently and predominantly focused on host or interphase engineering. Since the primary objective of LMBs is to exploit the ultra-high theoretical capacity of metallic lithium itself, POFs are mostly utilized as inactive matrices to accommodate and regulate metallic lithium plating/stripping, rather than acting as the primary bulk storage medium. Nevertheless, integrating redox-active groups into these host frameworks provides a dual-function benefit, where the framework coordinates lithium ions to lower nucleation barriers while expanding the interfacial transfer kinetics. Due to their immense structural and chemical diversity, different classes of POFs offer distinct engineering pathways. The highly crystalline architectures of COFs and MOFs offer precise, ordered nanochannels that homogenize ion transport and provide dense lithiophilic nucleation sites. Meanwhile, COPs and other emerging frameworks supply flexible, resilient, and electronically tunable extended networks to maintain robust interfacial contact. The following subsections categorize and critically review recent advancements in applying COFs, MOFs, COPs, and other POFs, highlighting how their tailored material properties directly translate into enhanced Coulombic efficiency, reduced polarization, and prolonged cycling lifespan in advanced lithium-metal batteries.

### 3.1. Covalent Organic Frameworks

For lithium-metal anode applications, the defining advantage of COFs lies in their highly ordered, periodic, and tunable nanochannels. These predictable pathways facilitate rapid lithium-ion transport and significantly homogenize the interfacial ion flux. Furthermore, the molecular backbones of COFs can be precisely engineered with diverse functional groups and heteroatoms to create abundant lithiophilic active sites. This synergistic combination of ordered physical confinement and strong chemical affinity effectively lowers the nucleation barrier, accelerates ion desolvation, and guides uniform lithium deposition. Consequently, COFs have been extensively explored as advanced artificial SEI and structural hosts to suppress dendrite proliferation fundamentally and enhance the reversibility of lithium plating and stripping during prolonged cycling.

**Figure 4:**
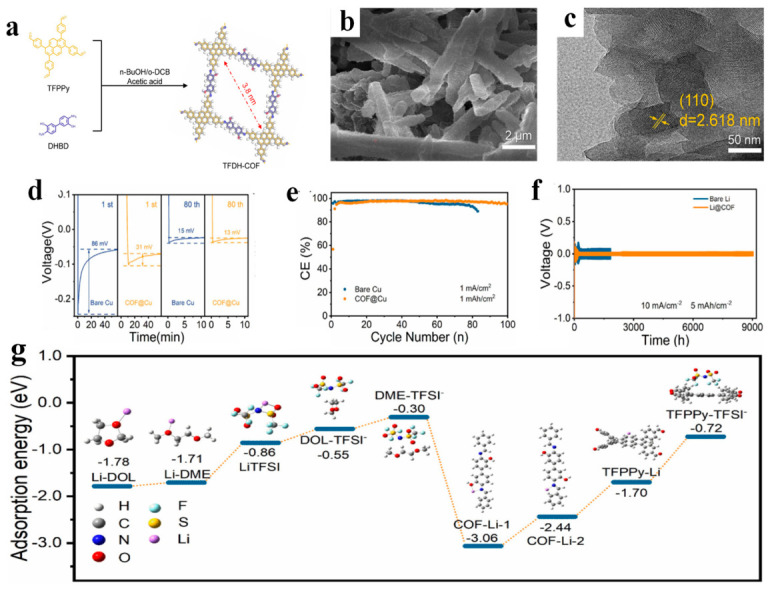
TFDH-COF: (**a**) Schematic diagram of lithium deposition, (**b**) SEM and (**c**) HR-TEM images, (**d**) nucleation overpotential, (**e**) Coulombic efficiency, (**f**) cycling curves at 10 mA cm^−2^ and 5 mAh cm^−2^ and (**g**) profiles of simulated adsorption energy viewed from Li^+^ with different solvent species and TFDH-COF fragments. Reproduced with permission from ref. [55] Copyright 2025, Elsevier.

Gou et al. developed a highly crystalline, nanorod-shaped pyrene-based TFDH-COF artificial SEI to facilitate Li^+^ regulation and suppress dendrite growth on lithium metal [55]. The TFDH-COF was synthesized via Schiff-base condensation of TFPPy and DHBD to form an ordered framework (Figure 4a). Uniform rod-like particles with diameters of 400–700 nm and clear lattice fringes of 2.618 nm corresponding to the (110) plane confirmed high crystallinity and 1D nanochannels (Figure 4b,c). The nucleation overpotential (Figure 4d) decreased from 86 mV (bare Cu) to 31 mV (COF@Cu) at 1 mA cm^−2^ and 1 mAh cm^−2^. CE of the half-cell fell below 90% after 84 cycles for bare Cu but averaged 97.14% over 104 cycles for COF@Cu (Figure 4e). Voltage hysteresis reached 64, 116, and 130 mV with lifespans of 8341, 11,585, and 3536 cycles at 10 and 20 mA cm^−2^ under 5 and 10 mAh cm^−2^, whereas bare Li showed 160, 480, and 422 mV and failed after 1691, 2592, and 1241 cycles (Figure 4f). Strong Li^+^ adsorption was evidenced by binding energies of −3.06 eV (COF-Li-1) and −2.44 eV (COF-Li-2), lower than −1.71 eV (Li-DME), −1.78 eV (Li-DOL), and −1.70 eV (pyrene-Li^+^), while TFSI^−^ showed −0.72 eV, and Li^+^ desolvation and migration energies were 50 and 20 kJ mol^−1^, respectively (Figure 4g). The highly crystalline, π-conjugated porous framework provided continuous 1D ion pathways and abundant lithiophilic functional groups, which accelerated desolvation and ion transport, homogenized interfacial flux, suppressed dendrite nucleation, minimized electrolyte decomposition, stabilized the solid electrolyte interphase, and maintained long-term structural integrity.

**Figure 5:**
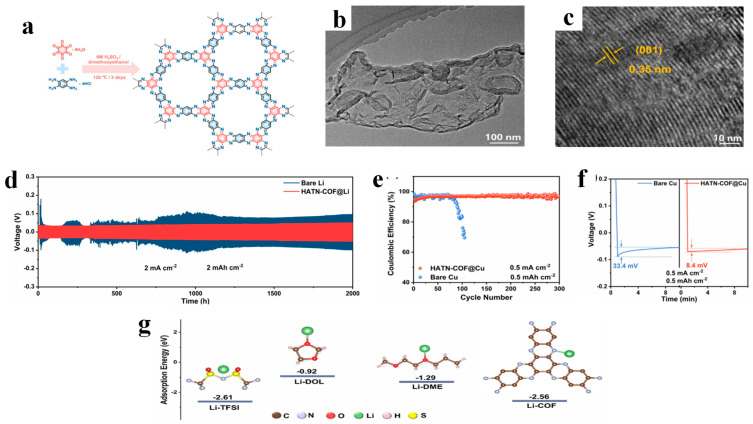
HATN-COF: (**a**) Schematic illustrations of the synthesis, (**b**) TEM and (**c**) HR-TEM images, (**d**) cycling stability at 2 mA cm^−2^ and 2 mAh cm^−2^, (**e**) Coulombic efficiency, (**f**) nucleation overpotential and (**g**) simulated chemical coordination and affinity energy. Reproduced with permission from ref. [56] Copyright 2025, Elsevier.

Shao et al. synthesized a fully cyclized aromatic HATN-COF as a highly crystalline artificial SEI to regulate Li^+^ transport and suppress dendrites in LMBs [56]. HATN-COF was synthesized from 1,2,4,5-benzene tetramine tetrahydrochloride and hexaketocyclohexane octahydrate in 6M H_2_SO_4_/dimethoxyethanol at 120 °C for 3 days, forming a periodic C=N linked porous framework (Figure 5a). Transmission Electron Microscope (TEM) and HR-TEM images revealed sheet-like morphology with a 100 nm scale and clear lattice fringes of 0.35 nm corresponding to the (001) plane and an interlayer spacing of 3.4553 Å, confirming high crystallinity and ordered channels (Figure 5b,c). The HATN-COF@Li symmetric cell operated over 2000 h at a current density of 2 mA cm^−2^ and areal capacity of 2 mAh cm^−2^ with a low hysteresis of 34 mV, outperforming bare Li (Li^+^ 0.68 vs. 0.56) (Figure 5d). In Li-Cu cells at 0.5 mA cm^−2^, bare Cu failed after 90 cycles, whereas HATN-COF@Cu exceeded 300 cycles with 96.85% CE (Figure 5e) and reduced nucleation overpotential from 33.4 mV to 8.4 mV (Figure 5f). Density Functional Theory (DFT) showed adsorption energies of −2.61 eV (Li-TFSI), −2.56 eV (Li-COF), −1.29 eV (Li-DME), and −0.92 eV (Li-DOL), evidencing strong Li^+^ affinity and selective transport (Figure 5g). The fully conjugated crystalline framework provided dense lithiophilic sites and vertically aligned nanochannels that promoted selective ion transport, accelerated desolvation, homogenized ion flux, suppressed parasitic reactions, stabilized the interphase structure, and guided uniform lithium deposition during prolonged cycling.

**Figure 6:**
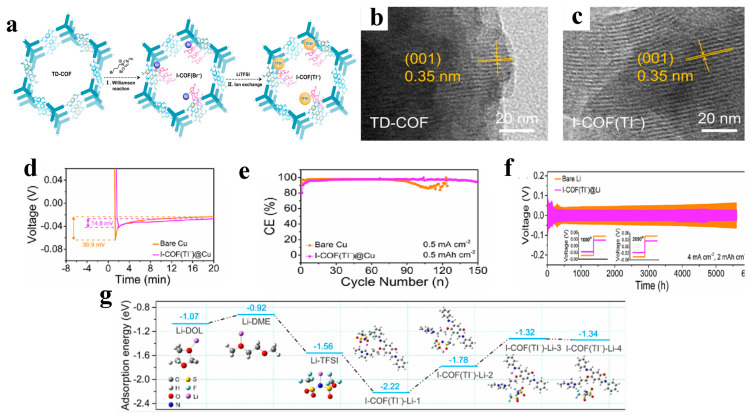
I-COF(TI^−^): (**a**) synthesis process, (**b**,**c**) HR-TEM images, (**d**) nucleation overpotential, (**e**) Coulombic efficiency, (**f**) cycling stability at 4 mA cm^−2^ and 2 mAh cm^−2^ and (**g**) profiles of simulated adsorption energy. Reproduced with permission from ref. [57] Copyright 2023, Elsevier.

Wang et al. engineered a highly crystalline TFSI^−^ ionic covalent organic framework (I-COF(TI^−^)) via spatial-partitioning to construct multi-dimensional Li^+^ transport nanochannels for stabilizing LMAs [57]. The conversion from 2D TD-COF to I-COF(TI^−^) through a Williamson ether reaction and LiTFSI anion exchange formed mixed-dimensional ionic channels (Figure 6a). Clear lattice fringes of 0.35 nm corresponding to the (001) plane with a 20 nm scale bar confirmed preserved crystallinity after modification (Figure 6b,c). The Li nucleation overpotential decreased from 39.9 mV on bare Cu to 14.8 mV on I-COF(TI^−^)@Cu (Figure 6d), while stable CE over 140 cycles at 0.5 mA cm^−2^ with 0.5 mAh cm^−2^ contrasted with bare Cu failure after 90 cycles (Figure 6e). Symmetric cells maintained stable cycling over 5500 h at 4 mA cm^−2^ (2 mAh cm^−2^) with 25.6 mV and operated at 10 mA cm^−2^ (5 mAh cm^−2^) with 52.1 mV, whereas bare Li short-circuited at 1240 h and initially showed 72.9 mV before 180 h, and stable rate performance from 0.25 to 6 mA cm^−2^ (Figure 6f). Li^+^ adsorption energies at I-COF-Li-1 and I-COF-Li-2 were −2.22 eV and −1.78 eV, stronger than TFSI^−^ (−1.56 eV), DOL (−1.07 eV), DME (−0.92 eV), and adjacent O/N sites (−1.32 eV), evidencing enhanced Li^+^ affinity and desolvation (Figure 6g). The spatially partitioned ionic nanochannels regulated uniform ion flux, strengthened lithiophilic interactions, restricted anion migration, stabilized interfacial chemistry, mitigated polarization, and mechanically confined lithium growth, enabling homogeneous deposition and highly reversible charge transfer.

**Figure 7:**
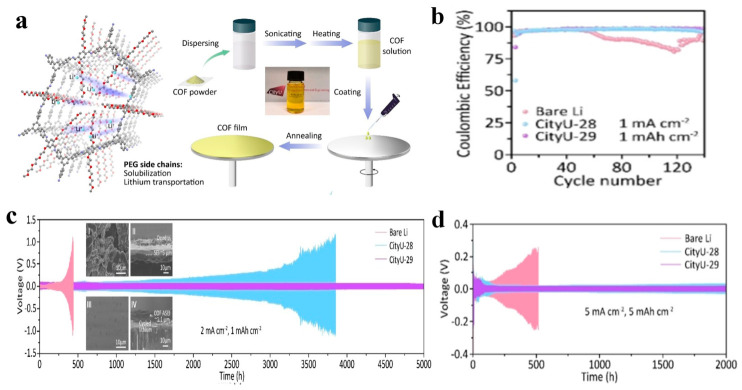
PEG: (**a**) schematic illustration, (**b**) Coulombic efficiency, (**c**) cycling stability at 4 mA cm^−2^ and 1 mAh cm^−2^ and (**d**) cycling stability at 5 mA cm^−2^ and 5 mAh cm^−2^. Reproduced with permission from ref. [58] Copyright 2025, Wiley-VCH GmbH.

Xu et al. developed soluble PEG-grafted COFs (CityU-28 and CityU-29) as lithiophilic artificial SEI layers to stabilize LMAs [58]. PEG side chains enabled solubilization and Li^+^ transport through COF pores, and the solution preparation involved dispersing, sonicating, heating, coating, and annealing (Figure 7a). The half-cell with CityU-29 sustained 98.5% CE after 150 cycles at 1 mA cm^−2^ and 1 mAh cm^−2^, compared to bare Li fluctuating after 40 cycles. The nucleation overpotential was reduced to 200 mV versus 420 mV (CityU-28) and 620 mV (bare), with an average CE of 99.52% over 50 cycles (Figure 7b). At a current density of 2.0 mA cm^−2^ and areal capacity of 1.0 mAh cm^−2^, bare Li showed 30 mV for 200 h and failed around 400 h, while CityU-28@Li (50 mV) and CityU-29@Li (80 mV) remained stable, with CityU-29@Li cycling over 5000 h and stabilizing at 65 mV; Scanning Electron Microscopy (SEM) after 100 cycles revealed dendrite-free dense deposition for CityU-29@Li (Figure 7c). At 5.0 mA cm^−2^ and 5.0 mAh cm^−2^, CityU-29@Li operated over 2000 h, CityU-28@Li over 820 h, and bare Li failed after 500 h, while at 0.5 mA cm^−2^ and 5.0 mAh cm^−2^ bare Li lasted 400 h versus over 1100 h for CityU-29@Li (Figure 7d). The PEG-grafted COF formed a uniform, robust, and ion-conductive interphase that homogenized Li^+^ flux, accelerated desolvation and pore-guided transport, prevented direct electrolyte contact, suppressed dendrite growth, and stabilized interfacial reactions during prolonged cycling.

**Figure 8:**
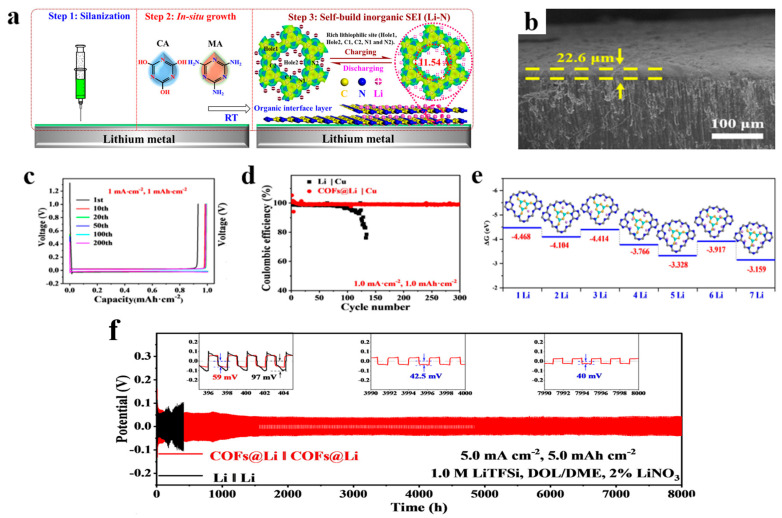
Nitrogen-rich triazine-based COF: (**a**) schematic diagram of the preparation process, (**b**) cross-section SEM image, (**c**) nucleation overpotential, (**d**) Coulombic efficiency, (**e**) DFT simulation and (**f**) cycling stability at 5 mA cm^−2^ and 5 mAh cm^−2^. Reproduced with permission from ref. [59] Copyright 2024, Royal Society of Chemistry.

Yue et al. constructed an in situ nitrogen-rich triazine-based COF on lithium metal to achieve a dendrite-free, ultra-stable anode [59]. A three-step selenization and in situ growth strategy formed a 2D COF with ordered 11.54 Å pores and multiple lithiophilic sites (Hole1, Hole2, C1, C2, N1, N2) for uniform Li^+^ regulation (Figure 8a). SEM image showed a dense COF layer of 22.6 μm tightly adhered to Li (Figure 8b). In Li@Cu cells at 1 mA cm^−2^/1 mAh cm^−2^, the nucleation overpotential decreased from 42 mV to 30 mV over 200 cycles (400 h) (Figure 8c), and CE retained 99.3% after 300 cycles, whereas bare Li failed after 122 cycles (Figure 8d). DFT revealed negative adsorption energy (ΔG_Li_) for fewer than 7 Li atoms, while the 8th Li migrated, confirming stable nucleation behavior (Figure 8e). Symmetric cells at 5 mA cm^−2^/5 mAh cm^−2^ cycled over 8000 h, and initial overpotentials of 19.8, 24.2, 28.6, 32.8, 35.4, 53.4, 65.8, and 73.2 mV at 0.5, 0.8, 1, 2, 5, 8, 10, and 20 mA cm^−2^ stabilized to 18, 21.8, 24.8, 30.4, 31, 47.2, 50.8, and 59.4 mV after 100 cycles, sustaining over 1600 h even at 20 mA cm^−2^/20 mAh cm^−2^ (Figure 8f). The nitrogen-rich lithiophilic framework, spontaneous Li-N interphase formation, robust mechanical confinement, and ordered channels homogenized Li^+^ flux and suppressed dendrite growth.

**Figure 9:**
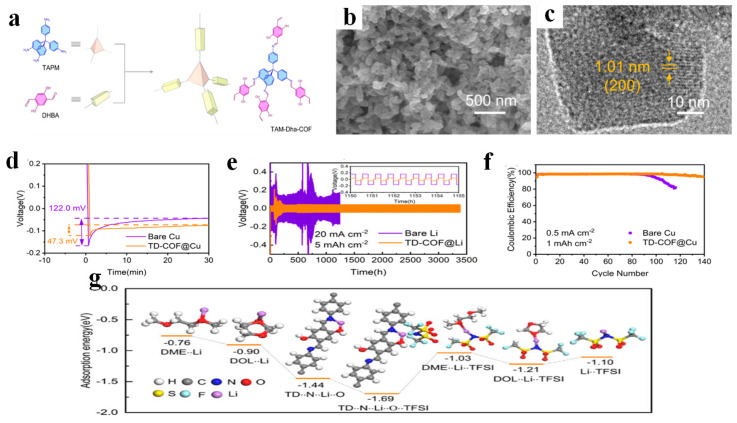
TD-COF: (**a**) synthesis process from TAPM and DHBA monomers, (**b**) SEM and (**c**) HR-TEM images, (**d**) nucleation overpotential, (**e**) cycling stability at 20 mA cm^−2^ and 5 mAh cm^−2^, (**f**) Coulombic efficiency and (**g**) DFT simulation. Reproduced with permission from ref. [60] Copyright 2024, Royal Society of Chemistry.

Zheng et al. developed a seven-fold interpenetrated dia-c7 3D TAM-Dha-COF (TD-COF) as an artificial SEI to stabilize LMAs [60]. TD-COF was synthesized via Schiff-base condensation of TAPM and DHBA, forming a 3D spatial framework, and lithium deposition with suppressed dendrites under spatial confinement was illustrated (Figure 9a). SEM image (Figure 9b) revealed ~200 nm octahedral particles and HR-TEM lattice spacing of 1.01 nm for (200) (Figure 9c). TD-COF@Cu exhibited 47.3 mV nucleation overpotential versus 122.0 mV for bare Cu, with hysteresis decreasing to 34.3 mV and 31.3 mV versus 38.2 mV and 68.6 mV for bare Cu (Figure 9d). Symmetric cells delivered stable overpotentials of 35.1 mV, 46.7 mV, and 80.7 mV with lifetimes of 7400 h, 9400 h, and 3300 h at 5, 10, and 20 mA cm^−2^ and operated from 0.25 to 6 mA cm^−2^ (Figure 9e). The half-cell maintained 95.05% CE after 140 cycles at 0.5 mA cm^−2^ and 1 mAh cm^−2^, >95% for 92 and 56 cycles at 1 and 2 mA cm^−2^, and >40 cycles at 3 mA cm^−2^ and 2 mAh cm^−2^ while bare failed within 2 cycles (Figure 9f). DFT showed Li^+^ binding energies of −1.44 eV and −1.69 eV with TD-COF versus −0.76 eV (DME), −0.90 eV (DOL), and −1.10 eV (TFSI^−^) which shows that the energetic Li^+^ interactions with TD-COF can facilitate the Li^+^ desolvation from the solvent cluster and expedite the adsorption on the nitrogen and oxygen sites of TD-COF. (Figure 9g). The interpenetrated 3D framework offered multidirectional ion channels and abundant lithiophilic sites that enhanced desolvation, homogenized ion flux, reduced polarization, stabilized LiF-rich interphases, and mechanically confined lithium growth to suppress dendrites.

**Figure 10:**
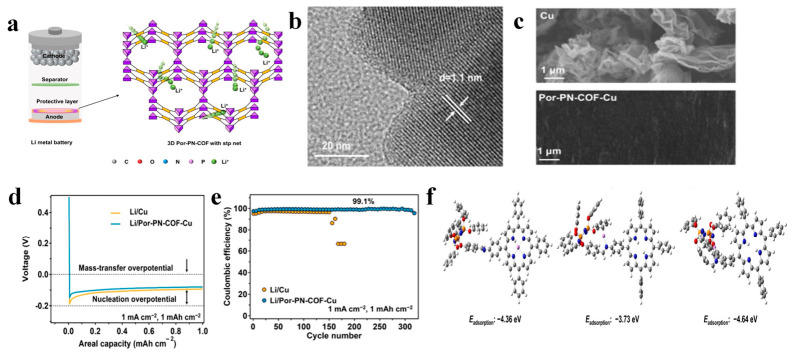
Three-dimensional Por-PN-COF: (**a**) schematic illustration of Li metal battery with Por-PN-COF protective layer, (**b**) HR-TEM and (**c**) SEM and LCSM image, (**d**) nucleation overpotential, (**e**) Coulombic efficiency and (**f**) DFT simulation. Reproduced with permission from ref. [61] Copyright 2024, Wiley-VCH GmbH.

Zheng et al. developed a Three-dimensional Por-PN-COF with dense lithiophilic heteroatoms as a protective layer for LMBs [61]. The 3D Por-PN-COF interlayer regulated Li^+^ flux in the battery configuration (Figure 10a), and HR-TEM revealed ordered stacking with a lattice spacing of 1.1 nm corresponding to the (310) plane (Figure 10b). Porous dendritic Li formed on bare Cu, while dense deposition occurred on Por-PN-COF-Cu at 1 mA cm^−2^ and 1 mAh cm^−2^ (Figure 10c). At 1 mA cm^−2^ and 1 mAh cm^−2^, the initial CE increased from 95.11% to 97.58% and nucleation overpotential decreased from 93.30 mV to 64.3 mV (Figure 10d); stable cycling reached 320 cycles with 99.1% average CE versus 153 cycles with 96.89% for bare Cu, and at 10 mA cm^−2^ and 2 mAh cm^−2^ delivered 95.4% CE over 82 cycles versus 93.6% over 40 cycles (Figure 10e). Strong Li^+^ binding energies of −4.36 eV, −3.73 eV, and −4.64 eV at sites 1–3 were observed compared with −6.77 eV (Li–TFSI), −1.93 eV (Li-DOL), and −2.19 eV (Li–DME) (Figure 10f). 3D framework offered abundant lithiophilic sites, accelerated desolvation and ion transport, homogenized Li^+^ flux, stabilized interfacial chemistry, and suppressed dendritic lithium growth during long-term cycling.

**Figure 11:**
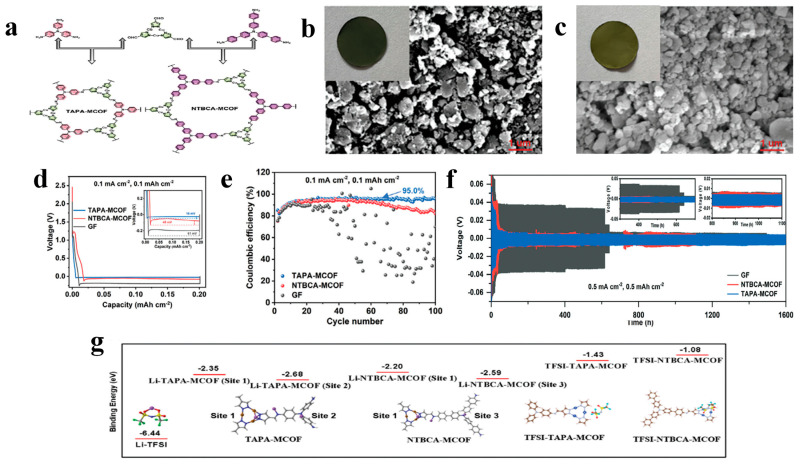
TAPA-MCOF and NTBCA-MCOF: (**a**) design principles for dendrite free LMBs, (**b**) SEM image of TAPA-MCOF, (**c**) SEM image of NTBCA-MCOF, (**d**) nucleation overpotential, (**e**) Coulombic efficiency, (**f**) cycling stability at 0.5 mA cm^−2^ and 0.5 mAh cm^−2^ and (**g**) DFT simulation. Reproduced with permission from ref. [62] Copyright 2025, Wiley-VCH GmbH.

Qin et al. designed redox-active COFs with dual-active trinuclear copper clusters and diarylamine units as functional separators for dendrite-free LMBs [62]. The solvothermal polycondensation between Cu_3_(PyCA)_3_·H_2_O and tris(4-aminophenyl)amine produced TAPA-MCOF and NTBCA-MCOF with dual polar centers (Figure 11a). The SEM images revealed that COF was distributed uniformly (Figure 11b,c). The Li nucleation overpotentials were 16 mV for TAPA-MCOF, 45 mV for NTBCA-MCOF, and 61 mV for GF (Figure 11d). In Li@Cu cells at 0.1 mA cm^−2^ and 0.1 mAh cm^−2^, the TAPA-MCOF achieved 95.0% CE after 100 cycles, whereas the GF cell showed an initial 76% and decayed after 22 cycles (Figure 11e). The bare Li cell exhibited an overpotential over 127 mV and a short-circuit failure after 620 h at a current density of 0.5 mA cm^−2^, indicating the growth of Li dendrites (Figure 11f). DFT results showed Li^+^ binding energies of −2.35 eV (Site 1) and −2.68 eV (Site 2) in TAPA-MCOF and −2.20 eV (Site 3) in NTBCA-MCOF, Li-TFSI dissociation energy of −6.44 eV, and TFSI^−^ adsorption energies of −1.43 eV (TAPA-MCOF) and −1.08 eV (NTBCA-MCOF) (Figure 11g). The dual redox-active centers created an electron-rich framework that enhanced salt dissociation, selectively coordinated lithium, immobilized anions, homogenized ion transport, stabilized interfacial chemistry, reduced polarization, and enabled uniform lithium nucleation and deposition during cycling.

The studies reviewed in this section collectively demonstrate that COFs provide a versatile platform for controlling Li^+^ transport and stabilizing LMAs, owing to their highly ordered porous structures and tunable chemical functionality. Early strategies focused primarily on crystalline 2D COF coatings, e.g., the pyrene-based TFDH-COF and HATN-COF, in which π-conjugated frameworks and vertical nanochannels enabled efficient Li^+^ migration and suppressed nucleation barriers, thereby improving CE and long-term cycling stability [60]. Subsequent work further highlighted the framework chemistry and the interactions of ions with the framework, wherein high Li^+^ binding energies and abundant lithiophilic sites in nitrogen-rich or aromatic structures facilitated enhanced Li^+^ adsorption and uniform nucleation. More advanced designs included functional and structural engineering, such as ionic COFs with spatially partitioned channels, PEG-grafted soluble COFs with enhanced interfacial contact and ion transport, and nitrogen-rich triazine frameworks that formed Li-N interphases to stabilize the interface [61]. In parallel, 3D interpenetrated COFs, such as TAM-Dha-COF and Por-PN-COF, demonstrated that multidirectional ion-transport channels and spatial confinement can further suppress dendrite growth and reduce polarization under high current densities. Functional COF separators with dual redox-active centers further demonstrated that integrating electrochemical functionality into the framework can control salt dissociation and selectively coordinate Li^+^. Overall, these studies reveal a clear evolutionary course in COF design from simple crystalline coatings to multifunctional architectures with lithiophilic chemistry, ion-selective channels, and structural confinement, all of which act in synergy to control Li^+^ flux, stabilize interfacial chemistry, and enable highly reversible Li^+^ deposition [62]. Consequently, COFs are a powerful class of materials for artificial SEI engineering, but additional efforts are still needed to optimize their mechanical robustness, electrolyte compatibility, and scalable synthesis to enable their practical implementation in high-energy LMBs.

### 3.2. Metal–Organic Framework

Metal–organic frameworks are highly tunable crystalline porous materials assembled from metal ions and organic ligands. In lithium-metal batteries, their ordered nanochannels and massive surface areas play a crucial role in homogenizing the Li^+^ flux. The intrinsic metal-centered active sites and polar functional groups within MOFs provide exceptional lithiophilicity, lowering the nucleation barrier and guiding uniform lithium deposition.

**Figure 12:**
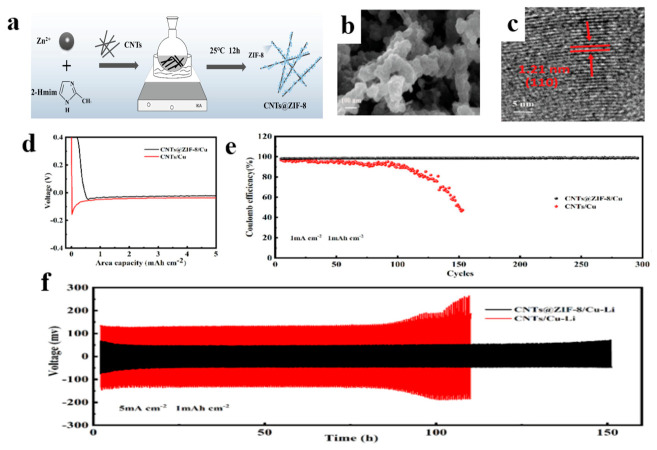
CNTs@ZIF-8: (**a**) schematic of preparation procedure, (**b**) SEM and (**c**) HR-TEM image, (**d**) nucleation overpotential, (**e**) Coulombic efficiency, (**f**) cycling stability at 5 mA cm^−2^ and 1 mAh cm^−2^. Reproduced with permission from ref. [63] Copyright 2023, Elsevier.

Sun et al. reported a 3D oxidized carbon nanotube framework decorated with nanosized ZIF-8 particles to regulate lithium nucleation and enable dendrite-free LMBs [63]. The CNTs were oxidized using mixed acid (H_2_SO_4_/HNO_3_ = 3:1) and reacted with Zn(NO_3_)_2_.6H_2_O and 2-methylimidazole in methanol at 25 °C for 12 h to form nano-ZIF-8 particles (Figure 12a). SEM image showed a regular porous 3D framework decorated with ZIF-8 nanoparticles (Figure 12b). HR-TEM displayed clear lattice fringes with an interplanar spacing of 1.21 nm corresponding to the (110) crystal plane of ZIF-8 (Figure 12c). Lithium nucleation overpotential on CNTs@ZIF-8/Cu was only 25 mV compared with 120 mV for CNTs/Cu at 1 mA cm^−2^, indicating a significantly reduced nucleation barrier due to the lithiophilic functional group (Figure 12d). CE tests at 1 mA cm^−2^ with an areal capacity of 1 mAh cm^−2^ showed that CNTs/Cu rapidly declined to below 40% after about 150 cycles, whereas CNTs@ZIF-8/Cu exhibited an initial CE of 98.77% and maintained around 98% for 300 cycles (Figure 12e). Symmetric cell testing at 5 mA cm^−2^ and 1 mAh cm^−2^ further revealed that CNTs@ZIF-8/Cu-Li maintained a stable voltage profile for over 100 h while CNTs/Cu-Li showed severe voltage fluctuations and micro-short circuits (Figure 12f). Combination of conductive nanotube networks and porous MOF nanoparticles provided abundant lithiophilic nucleation sites, enhanced electrolyte affinity, distributed ionic flux uniformly within the host framework, stabilized the electrode interface, and suppressed uncontrolled dendritic lithium growth during repeated plating and stripping.

**Figure 13:**
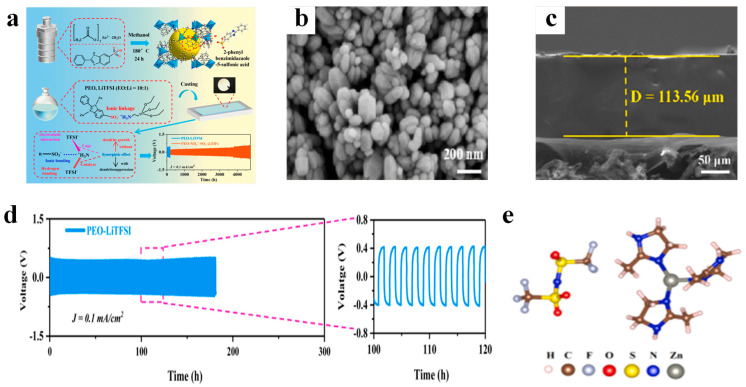
(**a**) schematic representation showing the preparation process, (**b**,**c**) cross-sectional SEM image, (**d**) time-dependent voltage profiles for symmetrical cells and (**e**) molecular structure of C_11_F_6_O_4_S_2_N_10_H_21_Zn. Reproduced with permission from ref. [64] Copyright 2023, Elsevier.

Zhou et al. reported a zwitter-ion modified MOF (NH_3_^+^·SO_3_^−^@ZIFs), which catalyzes LiF-rich SEI formation and suppresses lithium dendrites in LMBs [64]. The schematic design illustrated that NH_3_^+^·SO_3_^−^@ZIFs were constructed through ionic linkage between ammonium and sulfonate groups (Figure 13a). SEM characterization showed that ZIF particles possessed prismatic polyhedral morphology with particle sizes below 200 nm and were homogeneously embedded within the polymer matrix (Figure 13b). The cross-sectional structure of the PEO-NH_3_^+^·SO_3_^−^@ZIFs displayed a flat microstructure with an average thickness of 113.56 µm, indicating uniform filler dispersion (Figure 13c). Electrochemical evaluation using Li/Li symmetric cells at 60 °C and a current density of 0.1 mA cm^−2^ revealed that the cell suffered a short circuit after 180 h due to dendrite growth, whereas the Li/PEO-NH_3_^+^·SO_3_^−^@ZIFs/Li cell maintained stable cycling for approximately 300 h with reduced polarization, demonstrating effective dendrite suppression (Figure 13d). The molecular models depict the TFSI^−^ anion and the coordination environment around the zwitterionic NH_3_^+^·SO_3_^−^ modified ZIF and TFSI^−^, highlighting the atoms involved in electrostatic and hydrogen-bond interactions. These interactions regulate anion mobility and promote the formation of a LiF-rich SEI, thereby helping suppress lithium dendrite growth. (Figure 13e). Zwitter-ionic MOF structure immobilized anions promoted formation of a stable LiF-rich interphase, distributed lithium flux uniformly, stabilized electrode-electrolyte interfaces, and guided homogeneous lithium deposition during repeated cycling.

**Figure 14:**
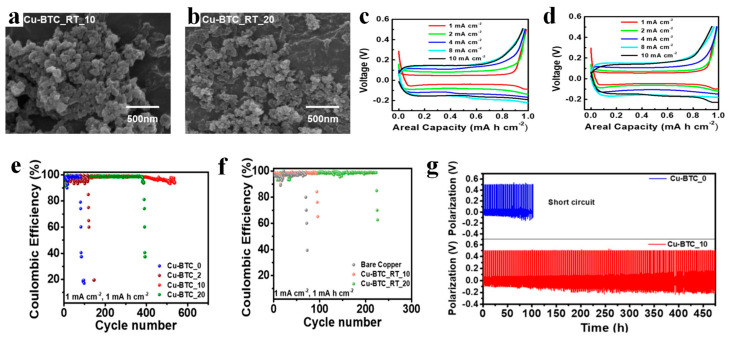
Cu-BTC-RT-10 and Cu-BTC-RT-20: (**a**) SEM image of Cu-BTC-RT-10, (**b**) SEM image of Cu-BTC-RT-20, (**c**,**d**) Nucleation overpotential, (**e**,**f**) Coulombic efficiency and (**g**) polarization profiles. Reproduced with permission from ref. [65] Copyright 2024, Royal Society of Chemistry.

Cheng et al. developed a modulated Cu-BTC (MOF) coating on copper foil to regulate lithium deposition and suppress dendrite growth, thereby enabling stable LMBs [65]. SEM analysis showed that both room-temperature-synthesized MOFs, Cu-BTC-RT-10 and Cu-BTC-RT-20, exhibited aggregated nanocrystalline particles with approximately 60 nm in size (Figure 14a,b). Rate-dependent lithium plating/stripping measurements showed that the Cu-BTC-10 electrode delivered superior Coulombic efficiencies of 99.1% at 1 mA cm^−2^ and 96.6% at 10 mA cm^−2^ compared with Cu-BTC-0, Cu-BTC-2, and Cu-BTC-20 electrodes because the microporous MOF modification layer homogenized Li^+^ flux during deposition (Figure 14c). Nucleation polarization analysis revealed that the Li nucleation overpotential decreased from 35.8 mV on bare copper to 8.0 mV after Cu-BTC-0 coating and further to only 0.6 mV with Cu-BTC-10 (Figure 14d). Electrochemical measurements at 1 mA cm^−2^ with a fixed capacity of 1 mAh cm^−2^ demonstrated that bare copper foil suffered rapid CE decay after about 65 cycles due to dendritic lithium growth, whereas MOF-modified electrodes showed significant improvement, maintaining cycling for about 95 cycles while Cu-BTC-RT-20 sustained approximately 220 cycles with 98% CE as the MOF specific surface area doubled (Figure 14e). Cu-BTC-0 showed CE decay after 80 cycles due to uneven coatings, Cu-BTC-2 extended the life to 120 cycles, Cu-BTC-20 achieved 300 cycles with 98% CE (Figure 14f). Long-term voltage profiles further showed that Cu-BTC-10 sustained stable operation for more than 450 cycles (Figure 14g). The nanoscale MOF modification layer regulated Li^+^ flux and promoted uniform lithium nucleation on the copper surface. Its high surface area, porous channels, improved electrolyte wettability, and reduced surface roughness enable homogeneous lithium deposition while suppressing dendrite formation and stabilizing the electrode-electrolyte interface during repeated cycling.

**Figure 15:**
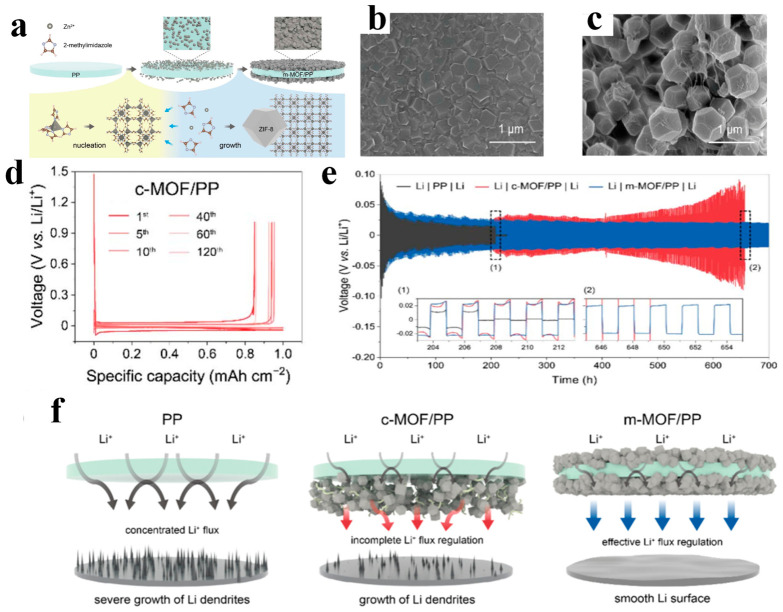
m-MOF/PP: (**a**) schematic illustration of the fabrication process, (**b**,**c**) SEM images, (**d**) nucleation overpotential, (**e**) cycling stability and (**f**) schematic illustration of Li^+^ flux through the PP, c-MOF/PP, and m-MOF/PP in contact with the Li metal anode. Reproduced with permission from ref. [66] Copyright 2024, Royal Society of Chemistry.

Kim et al. developed mechanically robust, void-free MOF monolith interlayers, directly grown on polypropylene separators, to regulate Li^+^ flux and suppress dendrite formation in LMBs [66]. The fabrication process involved nucleation of ZIF-8 by crystal growth forming a continuous monolithic MOF layer synthesized from precursor solutions (Figure 15a). Surface SEM analysis showed tightly packed rhombic dodecahedral grains with a grain size of approximately 200 nm and a uniform thickness of about 500 nm without interparticle voids (Figure 15b). The slurry-cast MOF layer contained discrete MOF particles of 500 nm bound by polymeric binder that generated continuous interparticle void networks (Figure 15c). Electrochemical Li plating-stripping tests using Li‖Cu cells at 0.5 mA cm^−2^ with an areal capacity of 1 mAh cm^−2^ delivered a CE of 83.2% after 130 cycles (Figure 15d). Symmetric cell tests at 1 mA cm^−2^ and 1 mAh cm^−2^ showed that cells experienced internal short-circuiting after 207 h corresponding to 101 cycles, whereas cells with m-MOF/PP maintained stable cycling for over 700 h corresponding to 342 cycles with uniform Li morphology (Figure 15e). The monolithic MOF interlayer with well-ordered nanoporous channels homogenizes Li^+^ flux at the electrode interface, enabling uniform lithium deposition and suppressing dendrite growth. In contrast, conventional PP and slurry-cast MOF layers lead to uneven Li^+^ distribution and dendritic lithium formation (Figure 15f). The monolithic nanoporous MOF architecture homogenized Li^+^ flux, filtered anions, eliminated interparticle void transport pathways, and provided a mechanically strong barrier that suppressed dendrite penetration while maintaining stable ion conduction and electrode morphology during repeated cycling.

**Figure 16:**
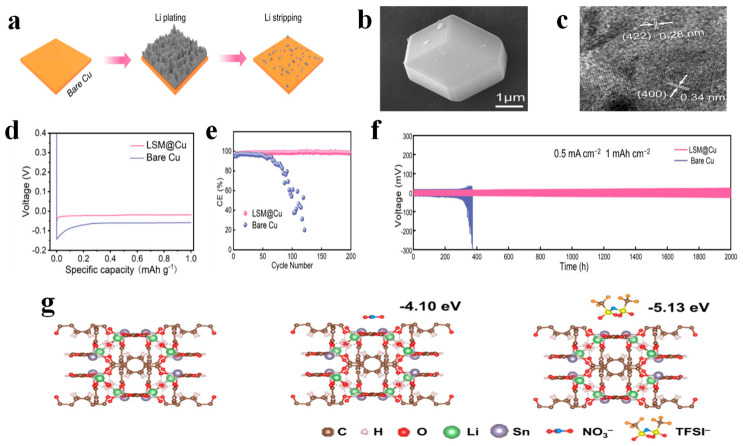
LSM@Cu: (**a**) schematic diagrams showing the Li plating/stripping, (**b**) SEM and (**c**) HR-TEM images, (**d**) nucleation overpotential, (**e**) Coulombic efficiency, (**f**) cycling stability at 0.5 mA cm^−2^ and 1 mAh cm^−2^ and (**g**) Schematic diagrams of NO_3_^−^ and TFSI^−^ anions attracted to the MOF. Reproduced with permission from ref. [67] Copyright 2024, Royal Society of Chemistry.

Chen et al. developed a Li_2_Sn_2_(bdc)_3_(H_2_O)_x_ MOF coating on Cu (LSM@Cu) that functioned as an artificial SEI to regulate Li^+^ flux and suppress dendritic lithium deposition [67]. Bare Cu caused uneven Li nucleation and dead Li formation due to lithiophobicity and tip amplification effects during plating/stripping (Figure 16a). SEM revealed polyhedral activated LSM particles with a size of 2 µm and a 1 µm scale bar (Figure 16b). HR-TEM displayed clear lattice fringes with spacings of 0.28 nm and 0.34 nm corresponding to the (422) and (400) plane (Figure 16c). The nucleation overpotential curve within a voltage window of −0.2 to 0.4 V and capacity range of 0–1.0 showed reduced polarization on LSM@Cu, indicating facilitated Li nucleation (Figure 16d). During cycling at 1 mA cm^−2^ with 1 mAh cm^−2^, LSM@Cu sustained an average CE of 98.6% for 200 cycles, whereas bare Cu dropped to 51.9% by 100 cycles (Figure 16e). In symmetric cells operated at 0.5 mA cm^−2^ and 1 mAh cm^−2^, LSM@Cu-Li maintained stable cycling for 2000 h with a small hysteresis of 25 mV, while bare Cu-Li showed rapid polarization increase to ~300 mV after 300 h due to dendrite formation (Figure 16f). DFT analysis indicated strong anion adsorption with binding energies of −4.10 eV and −5.13 eV at open metal sites, enabling anion immobilization and regulated Li^+^ transport (Figure 16g). MOF’s polar open-metal sites and ordered porous channels immobilized anions, homogenized Li^+^ flux, reduced nucleation barriers, and buffered volume changes, thereby enabling uniform lithium deposition and stable interfacial chemistry.

**Figure 17:**
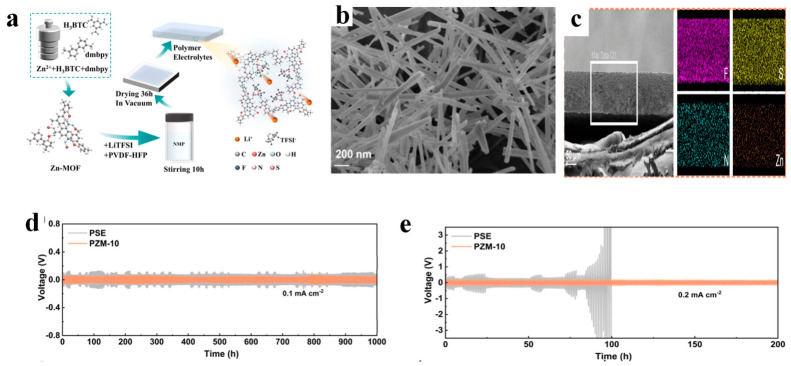
PZM-10: (**a**) Schematic of the preparation process, (**b**) SEM image, (**c**) EDS spectra and cycling stability at (**d**) 0.1 mA cm^−2^ and (**e**) 0.2 mAh cm^−2^. Reproduced with permission from ref. [68] Copyright 2025, Royal Society of Chemistry.

Zeng et al. developed a Zn-MOF incorporated PVDF-HFP/LiTFSI solid polymer electrolyte (PZM-10) to enhance Li^+^ transport and suppress dendrite formation in LMBs [68]. The synthesis schematic showed Zn-MOF formation from Zn^2+^, H_3_BTC and dmbpy followed by mixing with PVDF-HFP/LiTFSI using NMP, stirring for 10 h and vacuum drying for 36 h (Figure 17a). SEM image revealed Zn-MOF nanorods with a scale bar of 200 nm, confirming the formation of nanoscale MOF fillers suitable for incorporation into the polymer matrix (Figure 17b). EDS mapping displayed uniform distributions of F, S, N and Zn elements across the membrane, verifying homogeneous dispersion of PVDF-HFP, LiTFSI and Zn-MOF within PZM-10 (Figure 17c). Symmetric Li cells demonstrated stable cycling for over 1000 cycles at 0.1 mA cm^−2^ with significantly reduced polarization compared with PSE, indicating improved Li deposition behavior and interfacial stability (Figure 17d). At an increased current density of 0.2 mA cm^−2^, the Li/PSE/Li symmetric cell exhibited a rapid increase in overpotential and unstable voltage fluctuations within 100 cycles, indicating poor interfacial compatibility and dendritic lithium growth. In contrast, the Li/PZM-10/Li cell maintained stable voltage profiles for over 200 cycles with significantly lower polarization, demonstrating improved Li^+^ transport and effective dendrite suppression (Figure 17e). Zn-MOF reduced polymer crystallinity, promoted lithium salt dissociation and immobilized anions, enabling uniform Li^+^ transport pathways and stable interfacial chemistry that suppressed dendrite growth and improved cycling stability.

The studies discussed in this section emphasize the great potential of MOFs as multifunctional materials for stabilizing LMAs by structurally and chemically regulating lithium deposition. Their highly tunable porous architecture, high density of metal-centered active sites, and varied surface chemistries enable efficient control of Li^+^ nucleation and transport. Several options aim to incorporate MOF particles into conductive carbon frameworks, where the presence of porous structures and conductive networks provides multiple lithiophilic sites and ensures homogeneous current distribution, minimizing nucleation barriers and suppressing dendritic growth [66]. Other approaches use conductive composites from MOFs, metallic nanoparticles, and lithiophilic oxide domains to cooperatively control lithium nucleation and spatially distributed deposition, reducing local current density and stabilizing the electrode interface. Chemical functionalization of MOFs further enables interface regulation by immobilizing anions, facilitating the formation of stable LiF-rich solid electrolyte interphases, and limiting parasitic side reactions. Structural engineering strategies, such as microporous coating layers and monolithic MOF interlayers, provide ordered nanochannels that homogenize Li^+^ flux and serve as physical barriers against dendrite penetration [67]. In addition, incorporating MOF fillers into polymer electrolytes has been shown to enhance lithium salt dissociation, regulate ion transport pathways, and improve interfacial stability in solid-state systems. Collectively, these approaches demonstrate that MOFs can simultaneously control ion transport, stabilize interfacial chemistry, and mechanically confine lithium growth, enabling more uniform lithium deposition and improved cycling stability. Despite these promising advances, further efforts are still required to overcome intrinsic limitations, including the relatively low electrical conductivity in some pristine MOFs, potential structural degradation during prolonged cycling, and challenges associated with large-scale synthesis and integration into practical battery architectures [68].

### 3.3. Covalent Organic Polymer

Covalent organic polymers constitute an emerging class of protective interfacial materials characterized by extended π-conjugated backbones and flexible polymeric networks. Differentiating themselves from highly crystalline frameworks, COPs uniquely integrate electronic tunability with mechanical resilience. The incorporation of heteroatoms or single-metal sites into these conjugated structures establishes dense lithiophilic coordination environments that facilitate uniform lithium nucleation. Crucially, the inherent flexibility of these non-crystalline or semi-crystalline polymer networks profoundly impacts Li^+^ diffusion. In contrast to rigid crystalline frameworks where ions migrate exclusively through fixed, static nanochannels, the flexible chains of COPs can undergo dynamic conformational changes. This elastic deformation accommodates the massive volume expansion of deposited lithium and facilitates a polymer-chain-segmental-motion assisted ion transport mechanism. Such structural flexibility maintains the continuity of ion diffusion pathways even under high local mechanical stress, preventing the interfacial rupture and isolation of active sites commonly observed with rigid protective coatings.

**Figure 18:**
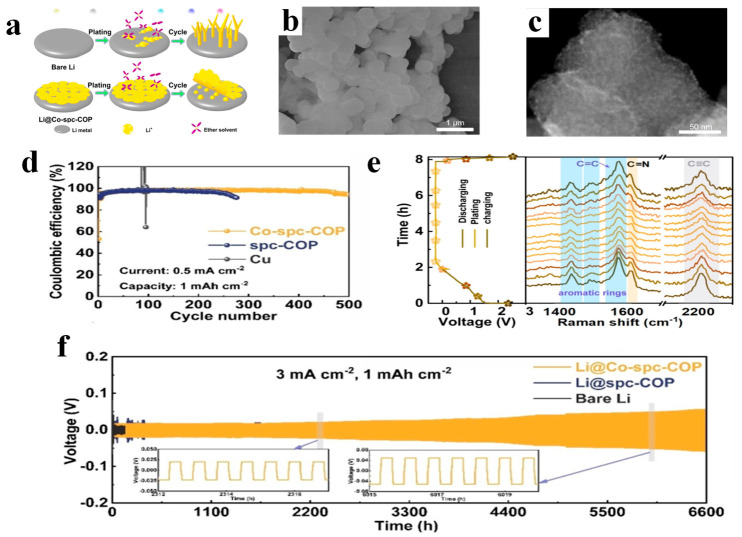
Co-spc-COP: (**a**) synthesis route of Co-spc-COP, (**b**) SEM and (**c**) HAADF-STEM images, (**d**) Coulombic efficiency, (**e**) in situ Raman spectra, and (**f**) cycling stability at 3 mA cm^−2^ and 1 mAh cm^−2^. Reproduced with permission from ref. [69] Copyright 2024, Wiley-VCH GmbH.

Lu et al. reported a cobalt-coordinated sp-carbon-conjugated organic polymer (Co-spc-COP) protective interfacial layer that regulated Li^+^ transport and suppressed dendrite formation in LMBs [69]. Electronic structure analysis showed that Co-spc-COP possessed a HOMO of −4.605 eV and LUMO of −3.273 eV compared with −5.577 eV and −2.948 eV for spc-COP, giving a reduced HOMO-LUMO gap of 1.332 eV that facilitated electron transfer and uniform Li deposition which is superior to that observed on bare Li electrodes (Figure 18a). Morphological characterization revealed uniform spherical Co-spc-COP particles with an average diameter of 80.0 nm (Figure 18b). Atomic-scale imaging further showed isolated Co atoms dispersed throughout the framework with short-range layered structures exhibiting ~0.37 nm interlayer spacing and nanosheet thickness of ~2.8 nm (Figure 18c). Electrochemical evaluation demonstrated that the Co-spc-COP electrode achieved a stable CE of 97.8% for 450 cycles at 0.5 mA cm^−2^ with an areal capacity of 1 mAh cm^−2^, outperforming spc-COP and bare Cu electrodes (Figure 18d). In situ Raman analysis identified reversible spectral variations at 2210 cm^−1^ (C≡C), 1615 cm^−1^ (C=N), and aromatic ring peaks at 1580 cm^−1^, 1500 cm^−1^, and 1430 cm^−1^ during lithiation and delithiation, confirming Li^+^ interaction with conjugated structures and indicating that lithium deposition occurred beneath the protective polymer layer (Figure 18e). Symmetric cells with Li@Co-spc-COP operated for 6600 h at 3 mA cm^−2^ and 1 mAh cm^−2^, whereas Li@spc-COP and bare Li sustained only 1500 h and 140 h, respectively, demonstrating greatly improved cycling stability and dendrite suppression (Figure 18f). The cobalt-coordinated sp-carbon conjugated polymer protective layer regulated interfacial chemistry and Li^+^ transport. Abundant functional groups and cobalt sites created strong lithiophilicity, promoted uniform ion distribution, enhanced electrolyte compatibility, and formed a mechanically robust interface, enabling stable lithium deposition while effectively suppressing dendrite growth and side reactions.

**Figure 19:**
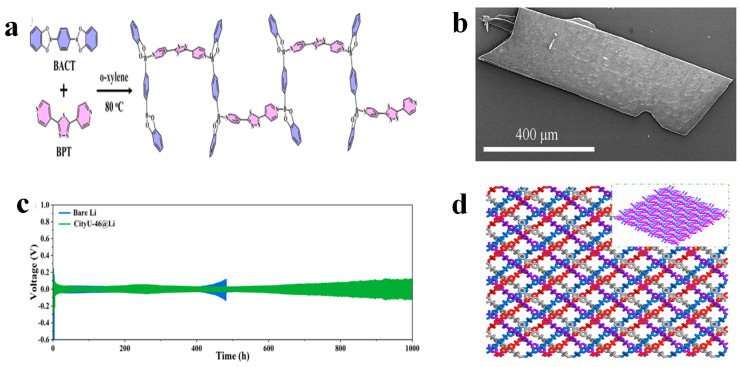
CityU-46: (**a**) synthetic route, (**b**) SEM image, (**c**) cycling stability at 1 mA cm^−2^, and (**d**) top view of a monolayer of the crystal packing in CityU-46. Reproduced with permission from ref. [70] Copyright 2025, Wiley-VCH GmbH.

He et al. reported the design of a two-dimensional, covalent organic polymer network (CityU-46) constructed via dative N→B bonds, serving as an artificial solid-electrolyte interphase to stabilize LMAs [70]. The synthesis involved linking 1,4-bis(benzodioxa-borole)benzene (BACT) and 2,5-bis(4-pyridyl)-1,3,4-thiadiazole (BPT) in a mixed solvent of methanol and o-xylene at 80 °C for 24 h followed by slow cooling (Figure 19a). Microscopic characterization revealed large crystalline plates where optical microscopy showed crystals around 500 μm and scanning electron microscopy displayed plate-like structures of about 400 μm (Figure 19b). Electrochemical evaluation showed that symmetric cells using CityU-46@Li operated at a current density of 1 mA cm^−2^ with an areal capacity of 1 mAh cm^−2^ maintained stable cycling for over 1000 h with a polarization voltage of approximately 18 mV after 90 cycles, whereas bare Li exhibited a higher polarization of 26 mV and failed after 400 h (Figure 19c). Single-crystal X-ray diffraction analysis demonstrated that four N→B-linked polymer strands interwove in the (100) plane forming a two-dimensional network with a two-over and two-under interweaving topology composed of warp-1, warp-2, weft-1, and weft-2 chains, stabilized by multiple noncovalent interactions including C–H–O interactions of 2.6, 2.7, and 3.1 Å, sandwich π–π interactions of 6.2, 6.3, and 6.5 Å, forming a robust woven molecular network (Figure 19d). The interwoven polymer architecture created a mechanically stable artificial interface that regulated ion transport pathways, enabled uniform lithium distribution, minimized localized current density, stabilized interfacial reactions, and guided controlled lithium deposition while suppressing dendrite formation during repeated cycling.

**Figure 20:**
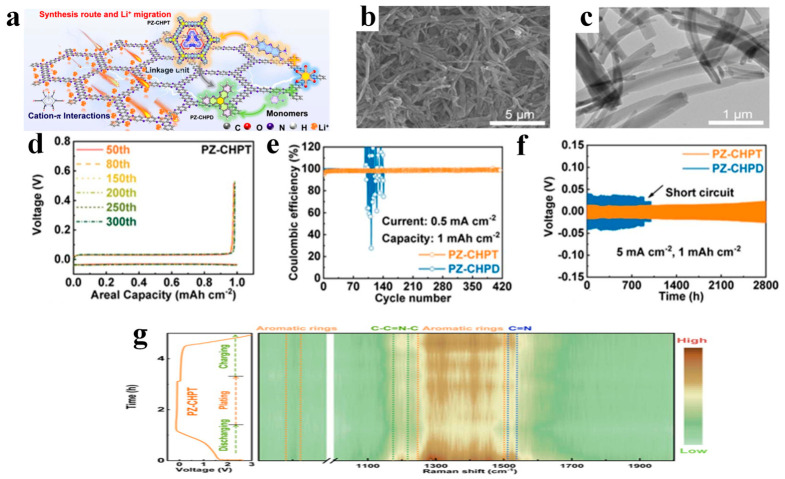
PZ-CHPT: (**a**) fabrication process and the Li^+^ migration mechanism, (**b**) SEM and (**c**) TEM images, (**d**) nucleation overpotential, (**e**) Coulombic efficiency, (**f**) Cycling stability at 5 mA cm^−2^ and 1 mAh cm^−2^ and (**g**) in situ Raman measurements and the associated voltage-time plots. Reproduced with permission from ref. [71] Copyright 2024, Wiley-VCH GmbH.

Lu et al. reported a ladder-type phenazine-linked covalent organic polymer (PZ-CHPT) protective interface designed to regulate Li^+^ transport through synergistic cation-π interactions and stabilize LMBs [71]. The structural concept illustrated the formation of ladder-type phenazine-linked covalent organic polymers derived from cyclohexanehexone and aromatic diamine units, generating an extended π-conjugated framework (Figure 20a). Although Figure 20a primarily depicts the local structural units, the rigid ladder-type polymer network formed by phenazine linkages spatially constructs a long-range π-π stacking and fully extended π-conjugated framework. Morphological characterization revealed that PZ-CHPD formed aggregated ribbon-like structures with diameters of approximately 200.0 nm (Figure 20b). The TEM image revealed that the PZ-CHPT polymer exhibited a well-defined nanorod morphology with uniform rod-like structures having an average diameter of approximately 30 nm (Figure 20c). The galvanostatic voltage profile demonstrated stable Li plating/stripping behavior in the Li‖PZ-CHPT@Cu cell, where the polarization voltage remained around 58.2 mV initially and increased slightly to 66.2 mV after 300 cycles (Figure 20d). Electrochemical Li plating/stripping tests demonstrated that Li‖PZ-CHPT@Cu delivered a stable CE of 98.7% for 415 cycles at 0.5 mA cm^−2^ with a capacity of 1 mAh cm^−2^, whereas Li‖PZ-CHPD@Cu failed after 97 cycles and exhibited CE below 63.9% after 103 cycles (Figure 20e). The galvanostatic cycling profiles demonstrated that the Li@PZ-CHPT‖Li@PZ-CHPT symmetric cell maintained stable lithium plating/stripping for over 2800 h at a current density of 5 mA cm^−2^ (Figure 20f). The in situ Raman spectra revealed the dynamic interaction between Li^+^ and the PZ-CHPT framework during plating and stripping. Characteristic vibrations assigned to C=N aromatic ring modes and C–C=N–C gradually weakened during discharge and recovered upon charging, indicating reversible Li^+^ coordination with the conjugated polymer (Figure 20g). Ladder-type conjugated polymer created a mechanically robust and electronically conductive interface that regulated Li^+^ distribution, stabilized interfacial reactions, enabled uniform nucleation and deposition, suppressed dendrite formation, and maintained stable ion transport pathways throughout prolonged cycling.

The studies discussed in this section show that COPs constitute a new class of interfacial materials for stabilizing LMAs by combining extended π-conjugated frameworks with tunable chemical coordination environments. Compared with crystalline COFs, COP-based systems focus on electronically conductive conjugated backbones and flexible polymeric architectures, which facilitate simultaneous control of Li^+^ transport and electron-transfer processes during Li^+^ plating and stripping [69]. One major strategy is to introduce metal-coordinated conjugated polymers, in which isolated metal atoms in sp-carbon conjugated frameworks alter the electronic structure, decrease the band gap, and provide a high density of lithiophilic coordination sites to ensure homogeneous Li nucleation and controlled deposition below the polymer layer [70]. Another design approach centers on structurally interwoven polymer networks, in which two-dimensional woven architectures stabilized by coordination bonds and noncovalent interactions form mechanically robust interfaces that uniformly distribute ion flux and resist dendrite penetration. More recently, ladder-type conjugated polymer frameworks with extended aromatic structures have been developed to exploit synergistic cation-π interactions, which enable reversible Li^+^ coordination with conjugated units and dynamic control of lithium plating/stripping processes [71]. Mechanistically, the polar heteroatoms (such as N and O) and extended π-conjugated systems embedded within these frameworks possess high electronegativity and electron-rich environments, establishing strong dipole-cation and cation-π interactions with the highly electron-deficient Li^+^. This strong Coulombic attraction effectively lowers the Li^+^ desolvation energy and the thermodynamic nucleation barrier, guiding uniform and dense lithium nucleation. Simultaneously, the robust mechanical confinement provided by the interwoven polymer network physically restricts the localized growth of lithium dendrites. These structural and chemical characteristics play an important role in boosting Li^+^ affinity, controlling ion migration routes, stabilizing interfacial reactions, and mechanically confining lithium growth, thereby reducing polarization, improving CE, and enhancing cycling stability under demanding working conditions. Overall, these studies indicate the emerging potential of conjugated COP frameworks as multifunctional artificial interphases for LMAs. However, additional studies are needed to enhance structural durability, optimize electronic conductivity, and devise scalable synthesis strategies for practical battery applications.

### 3.4. Other POF

Beyond conventional COFs, MOFs, and COPs, other emerging classes of porous materials, such as coordination polymers (CPs) and hydrogen-bonded organic frameworks (HOFs), have demonstrated unique advantages for stabilizing lithium-metal anodes. Driven by dynamic non-covalent interactions like multi-site hydrogen bonding and metal-ligand coordination, these frameworks offer highly adaptable and functionalized interconnected architectures. Their abundant polar sites are particularly effective at tailoring local solvation chemistry, anchoring free anions (such as TFSI^−^), and promoting the uniform distribution of Li^+^. By strategically regulating the ion transport environment, these alternative frameworks facilitate the formation of robust, inorganic-rich SEI and ensure highly reversible, dendrite-free lithium deposition.

**Figure 21:**
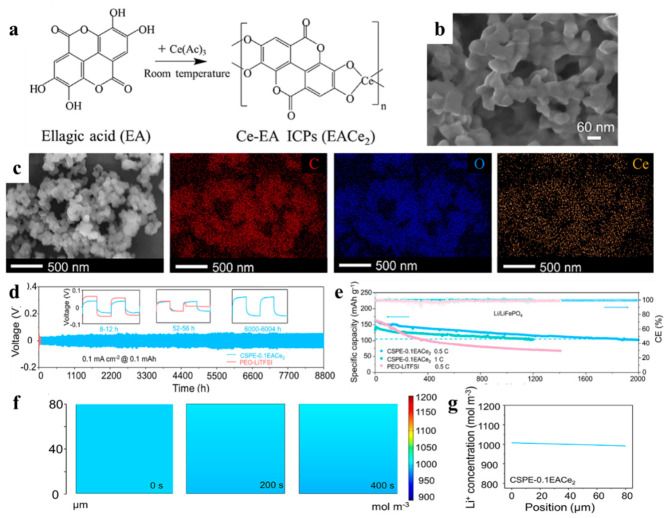
EACe_2_: (**a**) synthesis procedure, (**b**) SEM image, (**c**) EDS mapping images, (**d**) galvanostatic voltage profiles of Li/Li symmetric cells, (**e**) long-term cycling performance of all-solid-state Li/LiFePO_4_ full cells, (**f**) finite-element method simulations illustrating the Li^+^ diffusion behaviors and concentration gradients and (**g**) corresponding variation curve of the Li^+^ concentration. Reproduced with permission from ref. [72] Copyright 2021, Elsevier.

Wu et al. reported an amorphous infinite coordination polymer (ICP) nano-network composed of Ce-coordinate polyphenol ellagic acid (EACe_2_), which was introduced into a polyethylene oxide (PEO) matrix to develop composite solid polymer electrolytes (CSPE-EACe_2_) for highly endurable lithium metal batteries [72]. The synthesis process involved the self-assembly of EACe_2_ driven by the coordination of Ce^3+^ ions with the phenolic hydroxyl groups of ellagic acid (EA) at room temperature (Figure 21a). Morphological characterization via scanning electron microscopy revealed that the EACe_2_ ICPs formed an interconnected porous network consisting of block-like particles with an average diameter of approximately 60 nm (Figure 21b). The corresponding energy-dispersive X-ray spectroscopy mapping confirmed the uniform distribution of carbon, oxygen, and cerium elements, which excluded the possibility of phase segregation (Figure 21c). Galvanostatic cycling profiles of the symmetric cells demonstrated that the CSPE-0.1EACe_2_ membrane enabled highly stable lithium plating and stripping for over 8800 h at a current density of 0.1 mA cm^−2^ with an areal capacity of 0.1 mAh, effectively suppressing the early short circuits observed in bare PEO-LiTFSI electrolytes (Figure 21d). In all-solid-state full cell evaluations, the Li/LiFePO_4_ cells utilizing the CSPE-0.1EACe_2_ electrolyte delivered robust cycling stability and high reversibility, maintaining specific capacities of 102.6 mAh g^−1^ after 2000 cycles at 0.5 C and 103.2 mAh g^−1^ after 1200 cycles at 1 C, along with a Coulombic efficiency exceeding 99% (Figure 21e). Finite-element method simulations confirmed that the CSPE-0.1EACe_2_ electrolyte maintained a highly uniform Li^+^ concentration distribution with a minimal concentration gradient over time during ion migration (Figure 21f,g). Ultimately, the highly interconnected EACe_2_ filler network improved the Li^+^ transference number, alleviated cation concentration polarization. Mechanistically, the Ce^3+^ ions act as robust Lewis acid sites that can tightly coordinate with and anchor the mobile anions (such as TFSI^−^) from the liquid electrolyte. This anion-immobilization effect effectively restricts anion migration and smooths the concentration gradient, thereby promoting a uniform Li^+^ flux. Furthermore, the synergistic effect of the Ce^3+^ catalytic properties and the subsequent decomposition of the ellagic acid ligands facilitated the construction of a robust solid-electrolyte interphase enriched with LiF, Li_2_O, and Li_2_S, thereby promoting dendrite-free lithium deposition and maintaining stable ion transport throughout prolonged cycling.

**Figure 22:**
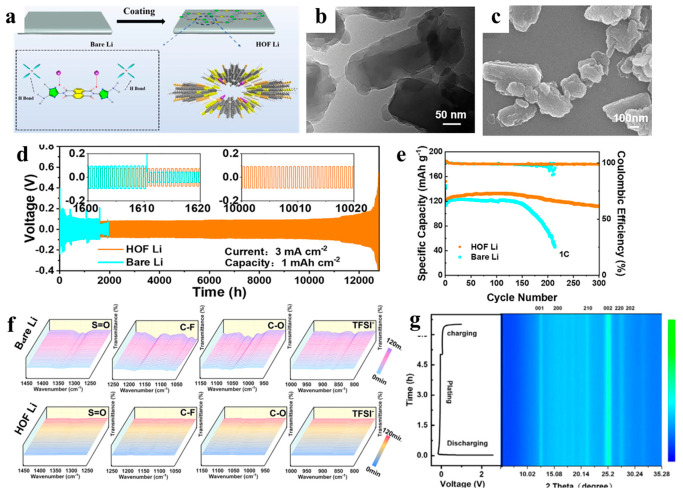
HOF-modified Li metal anodes: (**a**) schematic illustration of lithium accumulation and deposition kinetics on bare lithium, (**b**) TEM image of the two-dimensional HOF nanosheets, (**c**) SEM image, (**d**) long-term galvanostatic voltage profiles of symmetric cells, (**e**) cycling performance and Coulombic efficiency of Li/LiFePO_4_ full cells utilizing HOF Li and bare Li anodes, (**f**) in situ FT-IR spectra monitoring the evolution of electrolyte components during the charging and discharging processes and (**g**) in situ XRD patterns of the HOF electrode recorded continuously during the lithium plating and stripping stages. Reproduced with permission from ref. [73] Copyright 2025, Wiley-VCH GmbH.

Wu et al. reported a two-dimensional hydrogen-bonded organic framework (HOF) enriched with multi-site H-bonding and lithiophilic sites designed to tailor solvation chemistry and stabilize lithium metal anodes [73]. The structural concept illustrated the formation of HOF molecular sheets via an imidization reaction between 3,5-diamino-1H-1,2,4-triazole and 1,4,5,8-naphthalenetetracarboxylic dianhydride, providing abundant C=O and C=N lithiophilic sites alongside polar -NH_2_ groups (Figure 22a). Morphological characterizations including transmission electron microscopy and scanning electron microscopy revealed that the HOF consists of distinct two-dimensional sheet-like structures (Figure 22b,c). The galvanostatic cycling profile demonstrated extraordinary stability, with the HOF-protected Li symmetric cell maintaining stable lithium plating/stripping for over 11,000 h at a current density of 3 mA cm^−2^ with an areal capacity of 1 mAh cm^−2^ (Figure 22d). In full cell evaluations, the HOF-modified anode coupled with a LiFePO_4_ cathode exhibited superior long-term cycling stability and high Coulombic efficiency at 1 C (Figure 22e). In situ FT-IR spectra indicated that the HOF layer effectively suppressed the continuous decomposition of the electrolyte by anchoring the TFSI^−^ anions through hydrogen bonding (Figure 22f). Additionally, in situ X-ray diffraction patterns confirmed the highly reversible plating and stripping of lithium metal without the accumulation of “dead lithium” underneath the HOF layer during cycling, highlighting the excellent interfacial reversibility facilitated by the protective coating (Figure 22g).

The studies discussed in this section show that emerging porous materials such as coordination polymers and hydrogen-bonded organic frameworks have demonstrated unique advantages for stabilizing lithium-metal anodes. Driven by dynamic non-covalent interactions, these adaptable frameworks effectively anchor free anions, tailor local solvation structures, and promote the uniform distribution of Li^+^, facilitating dendrite-free lithium deposition. To systematically evaluate the advancements across these diverse materials, the critical electrochemical properties and cycling stability of various porous framework-modified lithium-metal anodes are compared. A comprehensive overview of their key performance metrics under different testing conditions is presented in Table 1.

### 3.5. Comparison of Framework Materials

While MOFs, COFs, and COPs share the common advantages of high porosity and structural tunability, their distinct physical and chemical natures dictate different trade-offs when applied to lithium-metal anodes. A critical comparison among these framework systems is essential for guiding rational material selection. A comprehensive comparison regarding lithiophilic sites and stabilization mechanism was made in Table 2.

MOFs are characterized by their ultra-high surface areas and abundant open metal sites. These metal nodes serve as excellent lithiophilic centers and Lewis acid sites, which strongly interact with electrolyte anions to promote a high Li^+^ transference number and facilitate uniform lithium nucleation. However, the practical application of MOFs is severely hindered by their extremely poor intrinsic electronic conductivity, which often leads to uneven electric field distribution during high-rate cycling. Additionally, the coordination bonds in MOFs are susceptible to chemical degradation in highly reducing environments or acidic electrolyte conditions [74].

COFs offer the highest pore precision and exceptional chemical stability due to their robust covalent linkages. The well-defined, uniform channels in COFs are highly effective in homogenizing the ion flux and physically blocking dendrite penetration. The primary drawback of these highly crystalline frameworks is their inherent mechanical rigidity. This stiffness limits their ability to dynamically buffer the massive volume fluctuations of the lithium metal anode during continuous plating and stripping cycles, potentially resulting in structural pulverization or interfacial delamination [75].

COPs provide a compelling alternative by balancing structural confinement with practical adaptability. COPs offer superior electronic tunability through extended π-conjugated systems, establishing efficient electron transport pathways. Furthermore, their flexible, often semi-crystalline or amorphous interwoven networks provide excellent mechanical elasticity. This flexibility allows COPs to accommodate the severe volume expansion of lithium metal and maintain intimate interfacial contact throughout prolonged cycling. The trade-off for this enhanced flexibility and conductivity is a lower degree of long-range order and less precise pore architectures compared to crystalline MOFs and COFs [76].

Ultimately, the optimal choice of framework material depends on the specific interfacial challenges being addressed: MOFs excel in ionic regulation via abundant active sites, COFs provide superior chemical stability and spatial confinement, and COPs offer the necessary mechanical resilience and electronic tunability for accommodating dynamic volumetric changes. From an architectural perspective, the choice between highly ordered crystalline structures (MOFs and COFs) and amorphous flexible networks (COPs) fundamentally dictates their electrochemical performance profiles. Ordered architectures feature low-tortuosity, periodic nanochannels that excel in delivering ultra-homogeneous ion flux and minimizing nucleation overpotentials, making them highly suitable for high-rate operations where ion transport kinetics are dominant. Conversely, amorphous networks lack long-range regularity but offer isotropic mechanical resilience and compliant interfacial contact, which effectively prevents structural pulverization over thousands of drastic volume expansion cycles. Based on recent literature outcomes, an emerging design principle is transitioning from single-component frameworks toward ordered-amorphous hybrids or crystalline-polymeric heterostructures, which synergistically combine the rapid ion-transport channels of ordered structures with the robust stress-dissipation capabilities of amorphous networks.

## 4. Design Principles of Porous Framework Anodes

To overcome these challenges, researchers have proposed several strategies to stabilize lithium metal anodes and improve LMB performance. One of the most widely explored approaches is electrolyte engineering, where engineers design advanced electrolyte systems capable of forming stable SEI layers and suppressing dendrite growth. For example, high-concentration electrolytes, solid-state electrolytes, and electrolyte additives have been studied to improve the lithium deposition behavior. Another approach is to construct artificial layers of SEI, or protective coatings, on the lithium surface [77,78,79]. These protective layers can help to enhance the mechanical stability of the SEI as well as to prevent direct contact between the lithium metal and the electrolyte. Materials, including polymers, inorganic compounds, and hybrid coatings, have been widely investigated for this purpose. More recently, the development of host materials or scaffold structures has become an extremely promising strategy for controlling lithium deposition. In this approach, porous and conductive materials serve as hosts to accommodate lithium metal during plating [80,81,82]. These host structures can help to reduce local current density, offer a large amount of nucleation sites for lithium deposition, and buffer the volume changes during the cycling process. Among host materials, porous frameworks such as MOFs, COFs, and COPs have attracted attention owing to their tunable structures, high surface areas, and functionalized frameworks [83,84]. Rational design of porous framework materials is extremely important for enhancing the stability and electrochemical performance of LMAs. These materials offer novel opportunities to regulate lithium nucleation, promote uniform lithium distribution, and ultimately enhance the safety and cycling stability of LMBs [85,86,87]. These characteristics allow them to control the nucleation and deposition behavior, as well as Li^+^ transport, within the electrode structure. However, to maximize such benefits and transition these materials from laboratory-scale half-cells to practical commercial applications, careful design strategies must be employed to optimize their structural, chemical, and electronic properties. Several important design principles have thus been developed to guide the development of porous framework anodes for LMBs, specifically tailored toward meeting critical performance requirements such as high areal capacities, stable operation under lean electrolyte conditions, and compatibility with ultra-thin lithium foils [88,89].

### 4.1. Structural Design and Pore Engineering

One of the most important design considerations for porous framework anodes is to optimize their pore structure. The porous architecture is important for controlling Li^+^ transport, providing nucleation sites for Li^+^ deposition, and accommodating volume changes associated with plating and stripping during repeated cycling. Materials with high porosity and large surface areas can distribute Li^+^ more evenly within the host structure, thereby reducing localized current density and suppressing dendrite formation. The size and distribution of the pores significantly affect lithium storage behavior. Micropores provide numerous adsorption sites for Li^+^ ions, and mesopores facilitate rapid ion transport and electrolyte penetration. Macropores, on the other hand, have the potential to act as reservoirs to accommodate deposited lithium metal and buffer large volume changes during the cycle [90,91,92]. Therefore, hierarchical pore structures with micro-, meso-, and macropores are particularly advantageous for the applications in LMBs. Such hierarchical frameworks can simultaneously promote the diffusion of Li^+^, the accessibility of electrolytes, and structural stability during long-term cycling. However, for practical implementation, the total volume and mass of the porous framework layer must be strictly controlled (e.g., coating thicknesses typically <5 μm) to prevent the sacrifice of the overall volumetric and gravimetric energy densities of the cell. In addition to pore size, the spatial distribution of pores also affects lithium deposition behavior. Ordered pore channels, as in crystalline materials such as MOFs and COFs, provide well-defined pathways for ion transport and facilitate uniform lithium deposition. This structural regularity reduces the formation of localized lithium accumulation, thereby promoting more homogeneous lithium growth within the host framework [93,94,95].

### 4.2. Chemical Functionalization and Lithiophilicity

Another key concept in design is adapting the chemical composition of porous frameworks to increase their affinity for Li^+^. The introduction of heteroatoms or functional groups into the framework can dramatically affect the nucleation behavior of lithium and enhance the interaction of Li^+^ with the host material. Elements such as nitrogen, oxygen, sulfur, and boron are often used in framework structures because they have high polarity and can serve as lithiophilic sites. Crucially, heteroatom engineering plays a multi-functional role in simultaneously optimizing the Li affinity, specific capacity, electrical conductivity, and SEI formation of porous frameworks. First, regarding Li affinity, the integration of highly electronegative heteroatoms generates intense local dipole-cation or cation-π interactions, which effectively lowers the thermodynamic nucleation overpotential and guides homogeneous initial Li plating. Second, this dense molecular-level distribution of active sites provides abundant pathways for multi-site adsorption, thereby maximizing reversible specific capacity and preventing active lithium loss. Third, heteroatom substitution modulates the intrinsic electronic band structure of the organic frameworks (e.g., narrowing the HOMO-LUMO gap), which significantly boosts electronic conductivity and accelerates interfacial charge transfer kinetics. Finally, heteroatoms fundamentally alter SEI formation chemistry; by selectively anchoring free anions (such as TFSI^−^) and tailoring the local solvation sheath, they promote the decomposition of beneficial species to form a mechanically robust, inorganic-rich (such as LiF^−^ or Li_3_N-rich) solid electrolyte interphase [96,97,98]. Lithiophilic functional groups lower the nucleation energy barrier for lithium deposition, allowing more uniform lithium metal nucleation on the surface of the host material. This homogeneous nucleation prevents local lithium build-up and reduces the probability of dendrite formation. In MOF-based materials, the metal centers can also act as active nucleation sites, thereby promoting strong interactions with Li^+^. Similarly, in COFs and COPs, functional groups such as carbonyls, imines, amides, and heterocyclic units can be used to increase the lithium adsorption and control the uniform deposition of lithium. Chemical functionalization can also affect the composition and stability of the SEI layer. Functional groups within the framework may interact with the electrolyte components, thereby promoting the formation of a more stable and uniform SEI. This improved SEI stability can be highly effective in enhancing CE and cycling performance in LMBs, which is particularly critical when batteries are required to operate under practical lean electrolyte conditions (i.e., low electrolyte-to-capacity ratios) where electrolyte depletion is a primary cause of cell failure [99,100].

### 4.3. Electronic Conductivity Enhancement

Although porous frameworks are excellent structural materials for storing Li^+^, many suffer from low intrinsic electrical conductivity. Poor conductivity can impede electron conduction within the electrode and lead to non-uniform lithium deposition, thereby affecting electrochemical performance. Therefore, increasing the electrical conductivity of porous framework materials is an essential consideration for design. Several strategies have been developed to address this limitation. Using porous frameworks in combination with conductive materials such as graphene, carbon nanotubes or conductive polymers has been proven to be an effective way. These hybrid materials form interconnected conductive networks that facilitate rapid electron transport throughout the electrode. In addition, the development of intrinsically conductive COFs with long π-conjugated systems has attracted increasing attention. Similarly, for MOFs, incorporating redox-active ligands or mixed-valence metal nodes can significantly boost intrinsic electron transport without sacrificing porosity. By constructing these continuous electron transport pathways, the sluggish interfacial charge transfer kinetics can be fundamentally resolved, ensuring a uniform electric field distribution. Such frameworks allow electrons to flow more readily through the material, thereby enhancing electrochemical performance without decreasing structural order. Achieving such high intrinsic conductivity is a prerequisite for sustaining the high current densities (e.g., >3 mA cm^−2^) and high areal capacities demanded by commercial applications [101,102].

### 4.4. Lithiophilicity Engineering and Metal Decoration

Engineering the Lithiophilicity of Porous Frameworks is another effective way to control Lithium deposition behavior. Materials with high affinity for Li^+^ promote the formation of a homogeneous deposition of lithium and thus decrease the likelihood of the formation of dendrites. In addition to heteroatom doping, lithiophilicity can be improved by introducing metal nanoparticles/catalytic sites into the framework [103,104]. Metal nanoparticles, such as silver, gold, zinc, or magnesium, can serve as favorable nucleation centers for lithium deposition. These metal sites lower the energy barrier for nucleation of lithium and favor the homogenous growth of lithium in the host structure. As a result, lithium metal is more likely to be deposited around these lithiophilic regions rather than to form uncontrolled dendritic structures on the electrode surface. Furthermore, some of these frameworks are metal-decorated and exhibit catalytic properties, controlling the Li^+^ flux and deposition kinetics. This catalytic behavior may be important for uniform lithium growth for repeated charge–discharge cycles. When combined with the hierarchical porosity and high surface area, these lithiophilic modifications create an ideal environment for steady lithium deposition and improved cycling efficiency [105,106,107].

### 4.5. Structural Stability and Mechanical Robustness

Finally, the capability to maintain the structural integrity of the host framework in repeated cycles of Li^+^ plating and stripping is important for long-term battery performance. Lithium metal undergoes large volume changes during cycling, which can cause mechanical stress and structural breakdown of the electrode material. Therefore, porous frameworks to be used as lithium hosts must have sufficient mechanical strength and structural stability. Highly cross-linked frameworks, as in many COFs and COPs, provide strong covalent bonding networks that contribute to mechanical durability [108,109,110]. In addition, flexible or hierarchical architectures can further enhance mechanical stability by allowing volume changes without inducing structural collapse. Crucially, looking toward forward-looking commercialization, this mechanical robustness must also translate to practical manufacturing. The framework coatings must be compatible with industrial roll-to-roll processing and maintain strong adhesion to ultra-thin lithium foils (<20 μm) without cracking or delamination. By leveraging mechanical robustness and a controlled pore structure with functionalized surfaces, the porous framework anode can effectively stabilize lithium metal and significantly extend the cycle life of LMBs [111]. Overall, the rational design of porous framework materials through structural engineering, chemical functionalization, conductivity enhancement, and lithiophilicity control offers a powerful strategy for designing advanced LMAs. These design principles are not only for improved lithium deposition behavior but also for bridging the gap between laboratory research and the stringent performance metrics required for the commercial viability of next-generation high-energy LMBs.

### 4.6. Degradation Mechanisms of Porous Frameworks and Mitigation Strategies

Despite their immense potential, the long-term stability of porous organic frameworks (POFs) in lithium-metal batteries is often compromised by severe degradation mechanisms under harsh electrochemical conditions. First, the highly reducing environment of the lithium metal anode can trigger the reductive cleavage of organic linkers and the destruction of coordination bonds, leading to the irreversible collapse of the crystalline pore structures. Second, the dramatic volume fluctuations of the lithium metal during repeated plating and stripping induce enormous mechanical stress, causing structural pulverization, fatigue, or delamination of the rigid framework coatings from the current collectors. Finally, trace moisture or acidic by-products (such as HF generated from LiPF_6_ decomposition) in the electrolyte can chemically attack the metal nodes of MOFs or the fragile linkages of certain COFs, exacerbating framework dissolution and active material loss [112,113].

To mitigate these degradation issues and develop robust next-generation materials, researchers are exploring several advanced strategies. Introducing flexible polymeric binders or constructing interwoven framework-polymer composites can effectively buffer mechanical strain and maintain intimate interfacial contact during volume expansion. Furthermore, designing frameworks with highly stable coordination geometries—such as employing high-valence metal nodes (e.g., Zr^4+^, Ti^4+^) in MOFs—or integrating chemically resilient covalent linkages (e.g., fully aromatic, fluorinated, or triazine-based COF backbones) can substantially enhance their thermodynamic and chemical stability against parasitic side reactions. Future research must prioritize the structural durability of these host materials to ensure their commercial viability under practical battery operating conditions [114,115,116].

## 5. Challenges and Future Prospects

**Figure 23:**
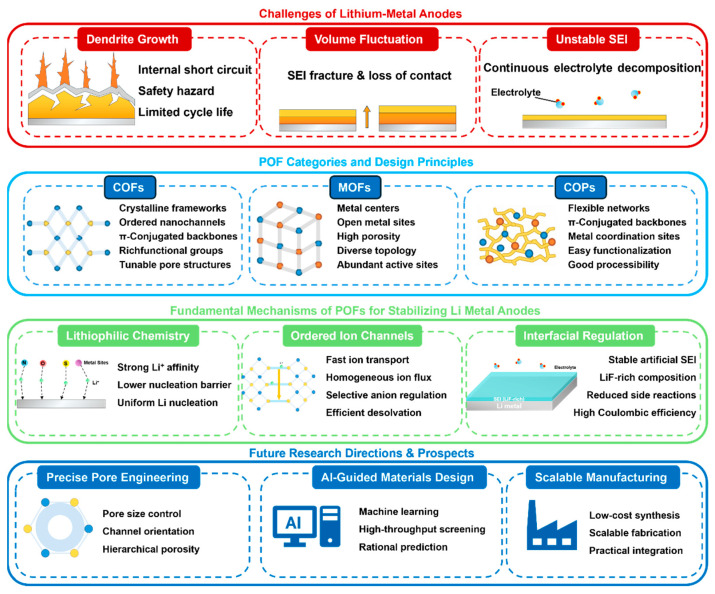
Schematic overview of the integrated design principles for porous framework anodes in LMBs.

Despite the substantial progress achieved in recent years, the transition of nanostructured porous framework materials, specifically COFs, MOFs, and COPs, from laboratory innovation to practical application in high-performance lithium metal batteries requires addressing several critical bottlenecks at the nanoscale. The following sections, as schematically summarized in Figure 23, outline the strategic research planning necessary to overcome current limitations and realize the full potential of these materials nanomaterials. The design of intrinsically conductive nanoframeworks remains the primary focus for future research. The low electrical conductivity of pristine COFs and MOFs is a major impediment to their widespread adoption, often leading to uneven lithium deposition and poor electrochemical performance at high current densities. While strategies such as hybridization with conductive carbon materials or converting MOFs into carbon frameworks have been explored, these methods often sacrifice the well-defined crystalline structure and chemical functionality of the original framework. Therefore, future research must prioritize molecular design to enhance intrinsic conductivity. For COFs, this involves developing frameworks with extended π-conjugated systems (e.g., 2D graphene-like architectures) and exploring pre-synthetic doping or generating radical intermediates to establish continuous electron transport pathways without compromising porosity. For MOFs, the focus should be on the implementation of redox-active ligands (such as tetrathiafulvalene derivatives) and mixed-valence metal nodes to boost electrical conductivity while maintaining structural diversity. Although COPs possess flexible polymer networks, their conductivity is often insufficient for high-rate applications; thus, designing highly conjugated COP networks is essential to bridge the gap between conventional polymers and high-performance conductive hosts.

Another critical area of development is the construction of hierarchical pore architecture. Optimizing ion transport kinetics and accommodating the massive volume fluctuations of lithium metal are vital for stable cycling. Future designs should integrate micro-, meso-, and macropores to achieve multiple objectives. To realize this, researchers should actively employ advanced synthetic protocols, such as template-directed synthesis (using block copolymers or colloidal spheres) and controlled post-synthetic etching techniques, to create predictable macroscopic void spaces. Ordered channels and micropores facilitate rapid Li^+^ diffusion and uniform nucleation, while mesopores and macropores provide the necessary space to accommodate deposited lithium and buffer volume changes during cycling. This well-designed pore structure helps reduce local current density, thereby suppressing dendritic growth. For COPs specifically, introducing well-defined hierarchical porosity will be crucial to overcoming the limitations imposed by their structural disorder and amorphous nature.

Enhancing structural stability and interfacial compatibility is also essential for long-term performance. The robustness of porous frameworks under harsh electrochemical conditions remains a significant challenge. Although COFs are chemically stable due to covalent bonding and COPs offer structural robustness, repeated lithium plating and stripping induce mechanical stress that can degrade the framework or its adhesion to the electrode. Furthermore, some MOFs are sensitive to moisture and electrolyte components, potentially leading to framework degradation. Poor adhesion or mechanical mismatch between the coating and the lithium metal surface can lead to interfacial degradation. Future research must focus on developing robust composite structures by integrating porous frameworks with flexible polymeric binders (e.g., self-healing elastomers) or constructing organic–inorganic interpenetrating networks to effectively dissipate localized mechanical strain. Furthermore, utilizing high-valence metal nodes in MOFs (e.g., Zr^4+^, Ti^4+^) and highly resilient linkage chemistry in COFs will be vital for resisting electrolyte corrosion. Understanding the interactions among metal nodes, electrolyte species, and lithium deposition behavior at the atomic level will be crucial for optimizing these interfaces.

Finally, scalability and cost-effectiveness are major hurdles for the commercialization of LMAs. The complex synthesis procedures and relatively high production costs associated with certain MOF and COF structures limit their viability for large-scale applications. Consequently, the development of scalable and cost-effective synthesis methods is imperative. Research must move beyond complex solvothermal procedures toward mechanochemical synthesis, continuous flow chemistry, and aqueous room-temperature co-precipitation. These greener, scalable methodologies are highly compatible with existing roll-to-roll manufacturing facilities, ensuring that these advanced materials can be seamlessly integrated into commercial pouch cell production lines. With continued advances in characterization and theoretical modeling, these materials will be instrumental in enabling safe, stable, and high-energy-density energy storage systems.

## Figures and Tables

**Table 1:** Electrochemical performance of POFs for LMBs.

Electrode	Current Density (mA cm^−2^)	Areal Capacity (mAh cm^−2^)	Cycling Hours (h)	Coulombic Efficiency (%)	Nucleation Overpotential (mv)	Ref.
TFDH-COF	10	5	9000	97.14	13	[56]
HATN-COF	2	2	2000	96.85	8.4	[57]
TD-COF	4	2	6000	98.12	14.8	[58]
CityU-29	5	5	2000	99.32	-	[59]
COF@Li	5	5	8000	99.24	30	[60]
TD-COF	20	5	3500	98.98	47.3	[61]
Three-dimensional Po-PN-COF	-	-	-	99.10	64.3	[62]
TAPA-MCOF	0.5	0.5	1600	95.0	16	[63]
CNTs@ZIF-8	5	1	150	99.1	9	[64]
PEO-NH_3_^+^⋅SO_3_0^−^ZIFs	0.1	-	4000	-	124.3	[65]
Cu-BTC-RT-10	1	1	450	96.6	8.0	[66]
m-MOF/PP	1	1	700	-	-	[67]
LSM@Cu	0.5	1	2000	98.6	-	[68]
Zn-MOF	0.1	0.2	1000	96.1	-	[69]
Co-spc-COP	3	1	6600	97.8	-	[70]
CityU-46	1	1	1000	-	-	[71]
PZ-CHPT	5	1	2800			[72]
EACe_2_	0.1	0.1	8800			[73]
HOF	3	1	11,000			[74]

**Table 2:** Summary of representative porous framework materials for stabilizing lithium-metal anodes.

Electrode	Framework Type	Lithiophilic Sites	Stabilization Mechanism	Ref.
TFDH-COF	COF	Pyrene-based π-conjugated framework	Homogenize ion flux and accelerate desolvation	[55]
HATN-COF	COF	C=N linked aromatic framework	Selective transport and interphase stabilization	[56]
TD-COF	COF	Spatially partitioned ionic channels, O/N sites	Anion restriction and mechanical confinement	[57]
CityU-29	COF	Soluble PEG side chains	Prevent electrolyte contact and homogenize flux	[58]
COF@Li	COF	Nitrogen-rich triazine sites (C=N)	Li-N interphase formation and physical confinement	[59]
TD-COF	COF	Three-dimensional interpenetrated framework, abundant sites	Three-dimensional multidirectional transport and LiF-rich SEI	[60]
Three-dimensional Po-PN-COF	COF	Dense lithiophilic heteroatoms	Interface stabilization and flux homogenization	[61]
TAPA-MCOF	COF	Trinuclear Cu clusters, diarylamine units	Salt dissociation and anion immobilization	[62]
CNTs@ZIF-8	MOF	Polar groups from oxidized CNTs, pyridinic N, and pyrrolic N	Uniform flux distribution via 3D conductive host	[63]
PEO-NH_3_^+^⋅SO_3_0^−^ZIFs	MOF	Zwitterionic NH_3_^+^ and SO_3_^−^ groups	Anion regulation and LiF-rich SEI catalysis	[64]
Cu-BTC-RT-10	MOF	Ordered porous channels	Flux homogenization and wettability improvement	[65]
m-MOF/PP	MOF	Monolithic well-ordered nanopores	Anion filtration and robust mechanical barrier	[66]
LSM@Cu	MOF	Polar open-metal sites	Anion immobilization and volume expansion buffering	[67]
Zn-MOF	MOF	Zn-MOF nanorods	Salt dissociation and uniform ion transport	[68]
Co-spc-COP	MOF	Isolated Co sites, sp-carbon network	Fast electron transfer and robust interface	[69]
CityU-46	MOF	Dative N→B bonds, π–π interactions	Minimize local current and mechanical stability	[70]
PZ-CHPT	MOF	Polar N/O heteroatoms, π-conjugated systems	Lower desolvation energy and physical confinement	[71]
EACe_2_	Other(ICP)	Ce^3+^ Lewis acid sites	Anion immobilization and inorganic-rich SEI promotion	[72]
HOF	Other(HOF)	C=O, C=N, and polar -NH_2_ groups	Solvation tailoring and anion anchoring	[73]

## Data Availability

No new data were created or analyzed in this study. Data sharing is not applicable to this article.
